# Safety and Seroconversion of Immunotherapies against SARS-CoV-2 Infection: A Systematic Review and Meta-Analysis of Clinical Trials

**DOI:** 10.3390/pathogens10121537

**Published:** 2021-11-24

**Authors:** Kevin Sheng-Kai Ma, Chien-Chang Lee, Ko-Jiunn Liu, James Cheng-Chung Wei, Yuan-Ti Lee, Li-Tzu Wang

**Affiliations:** 1 Center for Global Health, Perelman School of Medicine, University of Pennsylvania, Philadelphia, PA 19104, USA; kevinskma@hep1.phys.ntu.edu.tw; 2 Graduate Institute of Biomedical Electronics and Bioinformatics, College of Electrical Engineering and Computer Science, National Taiwan University, Taipei 10617, Taiwan; 3 Department of Dentistry, Chung Shan Medical University and Chung Shan Medical University Hospital, Taichung 40201, Taiwan; 4 Department of Emergency Medicine, National Taiwan University Hospital, Taipei 10002, Taiwan; cclee100@gmail.com; 5 National Institute of Cancer Research, National Health Research Institutes, Zhunan Township, Miaoli County 35053, Taiwan; kojiunn@nhri.edu.tw; 6 Department of Allergy, Immunology and Rheumatology, Chung Shan Medical University Hospital, Taichung 40201, Taiwan; wei3228@gmail.com; 7 School of Medicine, Chung Shan Medical University, Taichung 40201, Taiwan; 8 Division of Infectious Diseases, Department of Internal Medicine, Chung Shan Medical University Hospital, Taichung 40201, Taiwan; 9 Department of Obstetrics & Gynecology, National Taiwan University Hospital & College of Medicine, Taipei 10002, Taiwan

**Keywords:** coronavirus disease 2019 (COVID-19), severe acute respiratory syndrome coronavirus 2 (SARS-CoV-2), heterologous immunity, active immunity, passive immunity

## Abstract

Clinical trials evaluating the safety and antibody response of strategies to manipulate prophylactic and therapeutic immunity have been launched. We aim to evaluate strategies for augmentation of host immunity against severe acute respiratory syndrome coronavirus-2 (SARS-CoV-2) infection. We searched clinical trials registered at the National Institutes of Health by 25 May 2021 and conducted analyses on inoculated populations, involved immunological processes, source of injected components, and trial phases. We then searched PubMed, Embase, Scopus, and the Cochrane Central Register of Controlled Trials for their corresponding reports published by 25 May 2021. A bivariate, random-effects meta-analysis was used to derive the pooled estimate of seroconversion and adverse events (AEs). A total of 929,359 participants were enrolled in 389 identified trials. The working mechanisms included heterologous immunity, active immunity, passive immunity, and immunotherapy, with 62.4% of the trials on vaccines. A total of 9072 healthy adults from 27 publications for 22 clinical trials on active immunity implementing vaccination were included for meta-analyses. The pooled odds ratios (ORs) of seroconversion were 13.94, 84.86, 106.03, and 451.04 (all *p* < 0.01) for vaccines based on protein, RNA, viral vector, and inactivated virus, compared with that of respective placebo/control treatment or pre-vaccination sera. The pooled ORs for safety, as defined by the inverse of systemic adverse events (AEs) were 0.53 (95% CI = 0.27–1.05; *p* = 0.07), 0.35 (95% CI = 0.16–0.75; *p* = 0.007), 0.32 (95% CI = 0.19–0.55; *p* < 0.0001), and 1.00 (95% CI = 0.73–1.36; *p* = 0.98) for vaccines based on protein, RNA, viral vector, and inactivated virus, compared with that of placebo/control treatment. A paradigm shift from all four immune-augmentative interventions to active immunity implementing vaccination was observed through clinical trials. The efficacy of immune responses to neutralize SARS-CoV-2 for these vaccines was promising, although systemic AEs were still evident for RNA-based and viral vector-based vaccines.

## 1. Introduction

Coronavirus disease 2019 (COVID-19), caused by the corresponding pathogen severe acute respiratory syndrome coronavirus 2 (SARS-CoV-2), has rapidly threatened global health and economic systems [1,2]. Belonging to the Coronaviridae family, SARS-CoV-2 contains a positive single-strand RNA genome of 29.8 to 29.9 kb, which encodes two replicase/transcriptase ORF1ab and six accessory proteins, as well as four structural proteins, including nucleocapsid (N), envelop (E), membrane (M) and spike (S) proteins, with the last being the protein crucial to infecting target cells via binding to the angiotensin-converting enzyme II (ACE2) [3]. As a highly contagious virus, transmission sources of SARS-CoV-2 include discharge or direct contact with droplets and fomites that contain virus particles through the mouth, nose, or eyes. As of 25 May 2021, according to the Johns Hopkins Centers for Civic Impact [4], there were 170,354,142 confirmed cases and 3,541,800 deaths, with a mortality rate of 2.08%. Among all registered regions, the United States had the most cases of COVID-19, with 33,200,765 confirmed cases and 593,419 deaths. To date, rising numbers of clinical trials on the safety and antibody response of strategies to manipulate prophylactic and therapeutic immunity have been launched.

Host immunity is one of the most effective defense mechanisms against severely infectious diseases through comprehensive regulations in the immune system. If COVID-19 is to be effectively controlled, the most pivotal measure would be the development of effective immunity via vaccination or direct transfer of immunity for prophylactic or therapeutic purposes. Generally, three pathways of immunity induction are classified: vaccination to induce heterologous immunity or active immunity, transfer of active humoral immunity, which is also called passive immunity, and direct manipulation of immunity known as immunotherapy [5]. Among them, heterologous immunity refers to the induction of cross-protection by training innate immunity via vaccination of irrelevant pathogens [6]. On the other hand, active immunity triggers adaptive immune responses that involve both cellular and humoral responses with vaccine-induced immunological memory, while passive immunity utilizes antibodies against pathogen spreading and infection [7]. Immunotherapy is originally designed for the treatment of cancer by enhancement of anti-tumor immune activity via transfer of immune activator/cells or blockage of suppressor signals, such as programmed cell death protein-1 (PD-1)/PD-L1 pathway [8].

The holy grail that numerous clinicians and scientists are striving for during the COVID-19 crisis has been the successful vaccine or direct transfer of protective immunity. The purpose of this article is therefore to introduce and discuss these ongoing clinical trials registered at the National Institutes of Health (NIH) (ClinicalTrials.gov) for a systemic review and perform a meta-analysis on safety and antibody response for published results from current trials.

## 2. Results

### 2.1. Systematic Review of Clinical Trials

**Figure 1:**
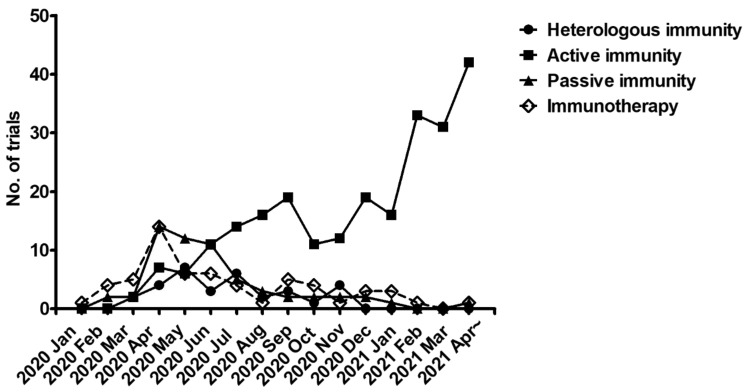
Accumulated numbers of clinical trials on prevention or therapy of COVID-19 via immunity augmentation by time-course analyses.

As of 25 May 2021, we appraised 389 registered clinical trials for COVID-19 based on non-pharmaceutical interventions (NPIS) for augmenting immunity, specifically vaccination or immunotherapy. A total of 929,359 healthy adults population susceptible to SARS-CoV-2 infection or patients with COVID-19 were enrolled in those clinical trials. Moreover, increasing numbers of registered trials aimed at inducing active immunity were observed in more current months (Figure 1). According to their working mechanisms, the 389 clinical trials may be classified into vaccination for induction of heterologous or active immunity, immunoglobulin transfer for providing passive immunity, and immunotherapy (Table 1, trial numbers; Table 2, participant numbers).

#### 2.1.1. Cross-Protective Vaccines Realizing Heterologous Immunity

Although they do not directly target SARS-CoV-2, the off-label use of *Mycobacterium* (phase 3: *n* = 19, participants = 29,202; phase 4: *n* = 6, participants = 10,864), measles mumps and rubella (MMR, phase 3: *n* = 2, participants = 260), poliovirus (phase 3: *n* = 1, participants = 3600; phase 4: *n* = 2, participants = 3425), and Zoster (phase 1: *n* = 1, participants = 250) vaccines have been rapidly authorized for clinical trials due to their well-established safety and potential to induce heterologous immunity. Among 26 trials using *Mycobacterium* vaccines, 20 of them used BCG, including 19 for prevention and one for therapy (trial number NCT04369794), in which humoral response against SARS-CoV-2 for the elimination of symptoms in patients with COVID-19. To determine whether BCG vaccination may prevent COVID-19 progression, we analyzed epidemiological data of reported COVID-19 cases by 12 September 2020, which was commonly used as a substitute when vaccination was not popularized. The retrieved data were from high-income countries whose claimed healthcare data on the BCG World Atlas [9] were considered reflective of the larger population (Supplementary Table S1). Although the incidence rate of COVID-19 was not different in countries with versus without BCG vaccination policy (Supplementary Figure S1A), the average mortality rate was significantly lower in countries implementing BCG vaccination policy (2.17%, ranging from 0% to 5.83%) than that of countries without such policy (5.1%, ranging from 0.73% to 12.56%) (Supplementary Figure S1B), suggesting the efficacy of BCG-mediated training or heterologous immunity for fir alleviating complications of COVID-19. Our cross-sectional analyses revealed significant differences in mortality for COVID-19 among countries with and without current BCG vaccination policies, indicating the protective role of BCG immunization on the induction of heterologous immunity against SARS-CoV-2. During the pandemic, off-label use of vaccines such as BCG and MMR vaccines [10] have been repurposed in the hope of establishing heterologous immunity against SARS-CoV-2 and have been provided to individuals with high-risk occupations for COVID-19, including healthcare providers.

#### 2.1.2. Inactivated and Subunit Vaccines Allowing for Active Immunity

Vaccination, as a process, could also induce active immunity or immunological memory, followed by a prophylactic effect on specific pathogens. Those defending the infected individuals through such a mechanism include killed or inactivated, toxoid, subunit, and live-attenuated vaccines. Among them, trials of killed or inactivated, and subunit vaccines against COVID-19 are still in early phases due to safety concerns. Trials aiming at inducing active immunity in healthy individuals include inactivated virus vaccines including inactivated virus (*n* = 42, total participants = 232,899) and S protein-derived subunit vaccines such as recombinant S protein vaccines (*n* = 49, participants = 172,232), mRNA vaccines (*n* = 68, total participants = 162,052), DNA vaccines (*n* = 13, participants = 8481), viral vector-based vaccines (*n* = 70, participants = 271,524), virus-like particles (*n* = 5, participants = 31,050), and live recombinant bacterial vectors (*n* = 1, participants = 84). Moreover, one of these trials evaluated the safety and antibody response of live-attenuated SARS-CoV-2 vaccines (participants = 48).

#### 2.1.3. Convalescent Plasma or Immunoglobulin Transfer Providing Passive Immunity

Among these trials aiming at transferring passive immunity, 18 trials administered intravenous immunoglobulin (participants = 2756), and 41 trials implemented convalescent plasma (participants = 13,864). In particular, 11 trials were in phase 3, and 1 trial was in phase 4, all of which revealed the therapeutic potential of immediate transfer of humoral immunity in COVID-19 patients.

#### 2.1.4. Immunotherapy

Immune checkpoint inhibitors such as PD-1 inhibitors and neutralizing antibodies have been widely used as immunotherapy agents [11]. Overreaction of the immune system has been reported to drive severe COVID-19 disease progression [12]. There were 29 trials (total participants = 3547) that evaluated immune checkpoint inhibitors including anti-PD-1 inhibitors such as nivolumab, anti-component 5a receptor (C5aR) monoclonal antibodies such as avdoralimab, and anti-IL6R neutralizing antibodies such as tocilizumab. Specifically, nivolumab was used to alleviate (cytotoxic T) T cell exhaustion that arose during SARS-CoV-2 infections [13], whereas avdoralimab and tocilizumab were expected to blockC5a/C5aR and IL6-IL6R pathways, which could bring about protective adaptive immunity [14] and block exuberant inflammation in COVID-19 pathogenesis [15]. On the other hand, immunotherapies aimed at increasing virus-eliminating immune pathways via the transfer of protective cytokines have been proposed. There were 14 trials (participants = 2329) that used cytokines such as IL-2 and IL-7 for lymphocyte activation [16] or type I IFNs to induce innate immunity against virus infections [17]. Ongoing trials on immune cell transfer (*n* = 16, participants = 1112) include the transplantation of lentivirus-modified DCs, antigen-loaded DCs, allogeneic natural killer (NK) cells, NK cells modified by CAR (NKG2D-ACE2 CAR-NK Cells), all of which were of phase 1 or 1&2, aiming at evaluating safety performance.

### 2.2. Meta-Analysis of Trial Reports

There were 9072 participants among the 27 studies included in the meta-analysis (Table 3). AEs in all studies were evaluated. The time from designated intervention to venipuncture for seroconversion was restricted within 28 days post-vaccination to verify that the immune response was rapid and specifically against the SARS-CoV-2 virus. The key characteristics and details of all included studies were described in Supplementary Table S2.

**Figure 2:**
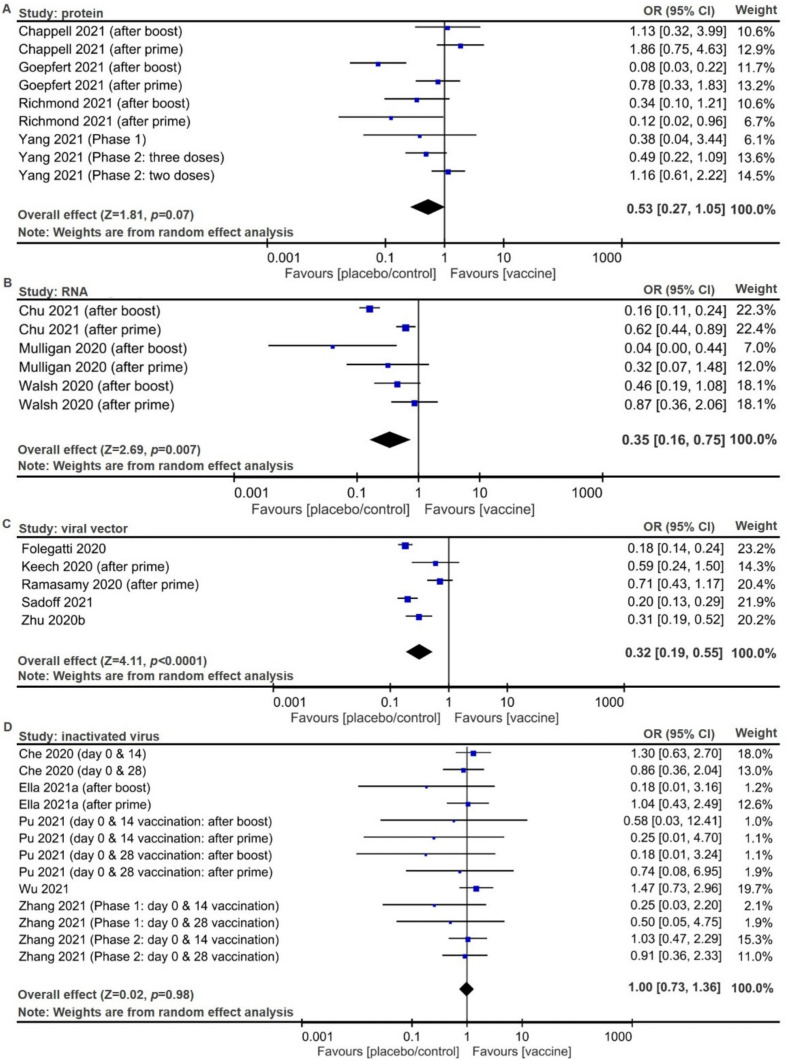
Forest plots for systemic adverse events (AEs) and summary estimates for safety of vaccines, defined as the inverse of sys-temic adverse events (AEs). Numbers of total participants and vaccinated populations with AEs for (**A**) protein vaccines, (**B**) RNA vaccines, (**C**) viral vector vaccines, and (**D**) inactivated vaccines. Random effect model was used to derive pooled inverse odds ratios (ORs) with 95% confidence intervals (CIs).

Overall safety of the subunit vaccines, defined as the inverse ORs of solicited sys-temic reactions, was derived for solicited systemic reactions of protein-based (Figure 2A, pooled OR 0.53, 95% CI 0.27 to 1.05; *p* = 0.07), RNA-based (Figure 2B, pooled inverse OR 0.35, 95% CI 0.16 to 0.75; *p* = 0.007), and viral vector-based (Figure 2C, pooled inverse OR 0.32, 95% CI 0.19 to 0.55; *p* < 0.0001) vaccines, and overall safety of inactivated vaccines were derived for inactivated virus-based vaccines (Figure 2D, pooled inverse OR 1.00, 95% CI 0.73 to 1.36; *p* = 0.98). Risks of solicited local reactions were derived for protein vaccines (Figure S2A, pooled inverse OR 0.12, 95% CI 0.06 to 0.24; *p* < 0.00001), RNA vaccines (Figure S2B, pooled inverse OR 0.04, 95% CI 0.02 to 0.07; *p* < 0.00001), viral vector vaccines (Figure S2C, pooled inverse OR 0.24, 95% CI 0.09 to 0.64; *p* = 0.04), and inactivated virus vaccines (Figure S2D, pooled inverse OR 0.46, 95% CI 0.29 to 0.72; *p* = 0.04) in which all four types of vaccine products can induce significant local AEs, compared with placebo/control. Risks of unsolicited AEs were derived for protein vaccines (Figure S3A, pooled inverse OR 0.90, 95% CI 0.60 to 1.34; *p* = 0.6), viral vector vaccines (Figure S3B, pooled inverse OR 0.48, 95% CI 0.30 to 0.77; *p* = 0.003), and inactivated virus (Figure S3C, pooled inverse OR 0.73, 95% CI 0.32 to 1.66; *p* = 0.46), while there was only one study addressing all unsolicited AEs for RNA vaccines [25].

Overall safety of the subunit vaccines, defined as the inverse ORs of solicited sys-temic reactions, was derived for solicited systemic reactions of protein-based (Figure 2A, pooled OR 0.53, 95% CI 0.27 to 1.05; *p* = 0.07), RNA-based (Figure 2B, pooled OR 0.35, 95% CI 0.16 to 0.75; *p* = 0.007), and viral vector-based (Figure 2C, pooled OR 0.32, 95% CI 0.19 to 0.55; *p* < 0.0001) vaccines, and overall safety of inactivated vaccines were derived for inactivated virus-based vaccines (Figure 2D, pooled OR 1.00, 95% CI 0.73 to 1.36; *p* = 0.98). Risks of solicited local reactions were derived for protein vaccines (Figure S2A, pooled OR 0.12, 95% CI 0.06 to 0.24; *p* < 0.00001), RNA vaccines (Figure S2B, pooled OR 0.04, 95% CI 0.02 to 0.07; *p* < 0.00001), viral vector vaccines (Figure S2C, pooled OR 0.24, 95% CI 0.09 to 0.64; *p* = 0.04), and inactivated virus vaccines (Figure S2D, pooled OR 0.46, 95% CI 0.29 to 0.72; *p* = 0.04) in which all four types of vaccine products can induce significant local AEs, compared with placebo/control. Risks of unsolicited AEs were derived for protein vaccines (Figure S3A, pooled OR 0.90, 95% CI 0.60 to 1.34; *p* = 0.6), viral vector vaccines (Figure S3B, pooled OR 0.48, 95% CI 0.30 to 0.77; *p* = 0.003), and inactivated virus (Figure S3C, pooled OR 0.73, 95% CI 0.32 to 1.66; *p* = 0.46), while there was only one study addressing all unsolicited AEs for RNA vaccines [25].

Overall safety of the subunit vaccines, defined as the inverse ORs of solicited sys-temic reactions, was derived for solicited systemic reactions of protein-based (Figure 2A, pooled OR 0.53, 95% CI 0.27 to 1.05; *p* = 0.07), RNA-based (Figure 2B, pooled OR 0.35, 95% CI 0.16 to 0.75; *p* = 0.007), and viral vector-based (Figure 2C, pooled OR 0.32, 95% CI 0.19 to 0.55; *p* < 0.0001) vaccines, and overall safety of inactivated vaccines were derived for inactivated virus-based vaccines (Figure 2D, pooled OR 1.00, 95% CI 0.73 to 1.36; *p* = 0.98). Risks of solicited local reactions were derived for protein vaccines (Figure S2A, pooled OR 0.12, 95% CI 0.06 to 0.24; *p* < 0.00001), RNA vaccines (Figure S2B, pooled OR 0.04, 95% CI 0.02 to 0.07; *p* < 0.00001), viral vector vaccines (Figure S2C, pooled OR 0.24, 95% CI 0.09 to 0.64; *p* = 0.04), and inactivated virus vaccines (Figure S2D, pooled OR 0.46, 95% CI 0.29 to 0.72; *p* = 0.04) in which all four types of vaccine products can induce significant local AEs, compared with placebo/control. Risks of unsolicited AEs were derived for protein vaccines (Figure S3A, pooled OR 0.90, 95% CI 0.60 to 1.34; *p* = 0.6), viral vector vaccines (Figure S3B, pooled OR 0.48, 95% CI 0.30 to 0.77; *p* = 0.003), and inactivated virus (Figure S3C, pooled OR 0.73, 95% CI 0.32 to 1.66; *p* = 0.46), while there was only one study addressing all unsolicited AEs for RNA vaccines [25].

Overall safety of the subunit vaccines, defined as the inverse ORs of solicited sys-temic reactions, was derived for solicited systemic reactions of protein-based (Figure 2A, pooled OR 0.53, 95% CI 0.27 to 1.05; *p* = 0.07), RNA-based (Figure 2B, pooled OR 0.35, 95% CI 0.16 to 0.75; *p* = 0.007), and viral vector-based (Figure 2C, pooled OR 0.32, 95% CI 0.19 to 0.55; *p* < 0.0001) vaccines, and overall safety of inactivated vaccines were derived for inactivated virus-based vaccines (Figure 2D, pooled OR 1.00, 95% CI 0.73 to 1.36; *p* = 0.98). Risks of solicited local reactions were derived for protein vaccines (Figure S2A, pooled OR 0.12, 95% CI 0.06 to 0.24; *p* < 0.00001), RNA vaccines (Figure S2B, pooled OR 0.04, 95% CI 0.02 to 0.07; *p* < 0.00001), viral vector vaccines (Figure S2C, pooled OR 0.24, 95% CI 0.09 to 0.64; *p* = 0.04), and inactivated virus vaccines (Figure S2D, pooled OR 0.46, 95% CI 0.29 to 0.72; *p* = 0.04) in which all four types of vaccine products can induce significant local AEs, compared with placebo/control. Risks of unsolicited AEs were derived for protein vaccines (Figure S3A, pooled OR 0.90, 95% CI 0.60 to 1.34; *p* = 0.6), viral vector vaccines (Figure S3B, pooled OR 0.48, 95% CI 0.30 to 0.77; *p* = 0.003), and inactivated virus (Figure S3C, pooled OR 0.73, 95% CI 0.32 to 1.66; *p* = 0.46), while there was only one study addressing all unsolicited AEs for RNA vaccines [25].

Overall safety of the subunit vaccines, defined as the inverse ORs of solicited sys-temic reactions, was derived for solicited systemic reactions of protein-based (Figure 2A, pooled OR 0.53, 95% CI 0.27 to 1.05; *p* = 0.07), RNA-based (Figure 2B, pooled OR 0.35, 95% CI 0.16 to 0.75; *p* = 0.007), and viral vector-based (Figure 2C, pooled OR 0.32, 95% CI 0.19 to 0.55; *p* < 0.0001) vaccines, and overall safety of inactivated vaccines were derived for inactivated virus-based vaccines (Figure 2D, pooled OR 1.00, 95% CI 0.73 to 1.36; *p* = 0.98). Risks of solicited local reactions were derived for protein vaccines (Figure S2A, pooled OR 0.12, 95% CI 0.06 to 0.24; *p* < 0.00001), RNA vaccines (Figure S2B, pooled OR 0.04, 95% CI 0.02 to 0.07; *p* < 0.00001), viral vector vaccines (Figure S2C, pooled OR 0.24, 95% CI 0.09 to 0.64; *p* = 0.04), and inactivated virus vaccines (Figure S2D, pooled OR 0.46, 95% CI 0.29 to 0.72; *p* = 0.04) in which all four types of vaccine products can induce significant local AEs, compared with placebo/control. Risks of unsolicited AEs were derived for protein vaccines (Figure S3A, pooled OR 0.90, 95% CI 0.60 to 1.34; *p* = 0.6), viral vector vaccines (Figure S3B, pooled OR 0.48, 95% CI 0.30 to 0.77; *p* = 0.003), and inactivated virus (Figure S3C, pooled OR 0.73, 95% CI 0.32 to 1.66; *p* = 0.46), while there was only one study addressing all unsolicited AEs for RNA vaccines [25].

**Figure 3:**
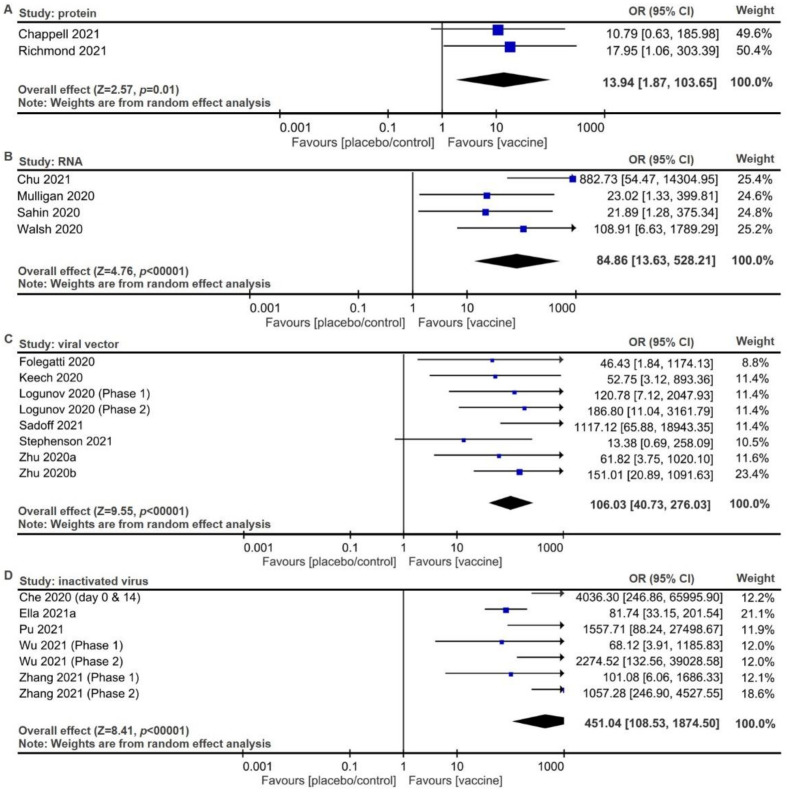
Forest plots for seroconversion to neutralize SARS-CoV-2 among adults inoculated with vaccines. Seroconversion was assessed within 28 days after vaccination of (**A**) protein vaccines, (**B**) RNA vaccines, (**C**) viral vector vaccines, and (**D**) inactivated vaccines. Random effect model was used to derive pooled ORs with 95% CIs.

Vaccination-mediated immune responses against SARS-CoV-2 were defined as seroconversion of at least a fourfold increase in the titers of neutralized antibodies against viral infection [45]. All vaccines can promptly induce seroconversion to block SARS-CoV-2 infection within 28 days post-vaccination. The seroconversion was derived for protein vaccines (Figure 3A, pooled OR 13.94, 95% CI 1.87 to 103.65; *p* = 0.01), RNA vaccines (Figure 3B, pooled OR 84.86, 95% CI 13.63 to 528.21; *p* < 0.00001), viral vector vaccines (Figure 3C, pooled OR 106.03, 95% CI 40.73 to 276.03; *p* < 0.00001), and inactivated virus vaccines (Figure 3D, pooled OR 451.04, 95% CI 108.53 to 1874.5; *p* < 0.00001). These findings suggest that both protein vaccines and inactivated virus vaccines are more tolerable and safer than RNA vaccines, followed by viral vector vaccines, and that inactivated vaccines have the highest efficacy to rapidly elicit serological responses, followed by viral vector vaccines, than RNA vaccines, and finally, protein vaccines based on their pooled ORs.

## 3. Discussion

In this systematic review of 389 clinical trials from the NIH Clinical Trial Database and meta-analysis of 27 published reports of the abovementioned trials, as well as one report for trials from the *Chinese Clinical Trial* Registry, an increasing number of immune-augmentative therapies for COVID-19 was observed. Moreover, the paradigm in this field has been gradually shifting from off-label use of irrelevant vaccines to active immunity induction against SARS-CoV-2, due mainly to their capabilities of providing specific protective immunity against SARS-CoV-2. In our systematic review, immuno-augmentative therapies presented promising immunogenicity and capabilities of reinforcing neutralized antibodies, which realized protective immunity against SARS-CoV-2 but at the same time addressed solicited systemic adverse reactions, solicited local adverse reactions, and unsolicited multiple organ adverse reactions.

With regard to training immunity against the novel pathogen SARS-CoV-2, previous studies have shown their capability to train adaptive immunity, which is immunological memory against specific pathogens [46]. That being said, the paradigm that immunological memory, if and only if it exists, in adaptive immunity has been constantly challenged due to the presence of pattern recognition receptors (PRRs), which are evolutionally conserved in most multicellular organisms and can specifically recognize infectious microbes via innate immune cells [47]. As the counterexample to the dogmatic concept, innate immune memory involving PRR-mediated pathways has been reported to cross-protect human bodies from irrelevant pathogens via epigenetic reprogramming in innate immune cells [48]. As a result, heterologous immunity plays a preventive role against novel pathogens that are critically threatening to humans without drugs or vaccines; this role is also known as trained innate immunity [49]. BCG immunization, originally designed for childhood tuberculosis, an infectious disease with high morbidity and mortality rates, has been one of the most prevalent vaccines [9]. Induction of nonspecific protection by BCG vaccination has been demonstrated in both animal and human studies [50,51], including several randomized-controlled trials (RCTs) [52,53]. For instance, BCG vaccination has been shown to cross-protect severe combined immune deficient (SCID) mice from disseminated candidiasis at a survival rescue from 30% to 100%, with T and B lymphocyte-independent pathways involved [50]. The induced cross-protection in BCG-vaccinated SCID mice was guided by activation of innate receptor nucleotide-binding oligomerization domain-containing protein 2 (NOD2) and epigenetic alterations at histone 3 lysine 4 trimethylation (H3K4me3) site in TLR-4 promoter regions, as well as other inflammatory cytokines in monocytes. Additionally, BCG vaccination has also been suggested to induce genome-wide epigenetic reprogramming of human monocytes and produce IL-1β against viremia caused by yellow fever virus infection in human studies [51]. In such a scenario, epigenetic reprogramming is considered a crucial mechanism of training innate immunity to develop immunological memory by regulating gene activation with histone modification, such asH3K4 methylation or H3K27 acetylation. Similarly, several RCTs have supported BCG vaccination-induced heterologous immunological effect on lowered morbidity and mortality among infants, as well as children suffering from infectious diseases other than TB [52,53]. Therefore, heterologous immunity realized with BCG vaccines has been considered as both a preventive and a therapeutic measure for SARS-CoV-2 infection [54].

A killed or inactivated vaccine refers to the injection of pathogens that have lost their disease-producing capacity but keeps the whole or partial structure consisting of multiple antigens that can be phagocytosed and digested by antigen-presenting cells (APCs), mainly dendritic cells (DCs), to realize adaptive immunity following subcutaneous or intramuscular administration. Then, antigen-loaded APCs via major histocompatibility complex type II (MHC II) molecules would migrate to secondary lymphoid organs to prime naive helper T (Th) cells, which helps antigen-bearing B cells differentiate to either memory B cells or short-lived plasma cells that first secret IgM, then undergo antibody class switching to produce IgG. Since the half-life of IgM is approximately 2 days, isotype switch to IgG would guarantee a prolonged half-life of around 20 days, ensuring the protective effect during infection [55]. In addition to providing multiple antigens, killed or inactivated vaccines are stable and safe but require several doses to elicit an efficient and broadly protective immunity with adjuvant supplements [56]. That being said, the disadvantages of killed or inactivated vaccines include by chance the inactivated pathogen having evolved surface components to escape immune surveillance that would still downregulate immune response during vaccination [57]. Overall, killed or inactivated vaccines have been considered as potential candidates for active immunization against emerging pathogens, for instance, SARS-CoV-2.

Subunit vaccines as a subtype of inactivated vaccines involve immunogenic peptides of pathogens that are designed to trigger APC-mediated T and B cell memory against infectious diseases after subcutaneous or intramuscular administration of multiple doses plus adjuvants. The corresponding process of inducing active immunity is similar to that of killed or activated vaccines. Candidate antigens in subunit vaccines are usually delivered by genetically engineered vectors, such as viral vectors or live bacterial vectors for recombinant DNA vaccines. In the case of coronavirus, S protein as a candidate antigen for both subunit vaccines and neutralizing antibodies would mediate the binding of host cell receptor ACE2 to viral pathogens, with the receptor-binding domains (RBD) of S proteins being similar in SARS-CoV-2 and SARS-CoV. Specifically, the S protein comprises two subunits, S1 and S2. The S1 subunit consists of an amino-terminal domain and an RBD. The RBD binds to ACE2 as its host cell target receptor, which starts the infection process. Therefore, subunit vaccines are capable of inducing antigen-specific-neutralizing antibodies that would target S proteins, hence preventing viral spread. These antigens presented in the FDA-approved clinical trials are commonly administered directly or using viral vectors including adenovirus, or bacterial vectors such as probiotics. Additionally, verified nanoparticle-based vaccines for respiratory viruses [58] are also assessed in the registered clinical trials, where the S antigen-encoded mRNAs contained in lipid-composed nanoparticles could directly be translated into functional S antigens after inoculation.

There are pros and cons of each vaccine design. For instance, DNA vaccines are not as immunogenic as mRNA or protein-based products [59], while mRNA vaccines are not as stable as DNA vaccines. Viral vector vaccines are usually more immunogenic than those using other vectors, yet viral vectors would bring about reduced efficacy due to preexisting immunity to the vector [60]. Subunit vaccines, focusing on S protein, the critical viral protein that binds to ACE2 on the host cells, are considered safer than live-attenuated vaccines and more specific than inactivated vaccines. Based on serological evidence through released studies, most protein vaccines, RNA vaccines, and inactivated vaccines required two doses to provide strong levels of seroconversion with ORs over 100, while viral vector vaccines can require only one dose to reach the comparably strong level of seroconversion (Table 3 and Figure 3). Storage requirements for protein vaccines, DNA vaccines, and inactivated virus vaccines include temperatures around 2 to 8 °C (36 to 46 °F), while that for RNA vaccines may vary among products, including (1) 2 to 8 °C (36 to 46 °F) for instant use or −20 °C (−4 °F) for long-term storage of mRNA-1723, and (2) −80 °C (−112 °F) for BNT162b1. Viral vector vaccines are suggested to be stored at 2 to 8 °C (36 to 46 °F) for instant use or −20 °C (−4 °F) for long-term storage (Table 3) [61].

Passive immunity refers to the transfer of humoral immunity, in which the involved protective immunoglobulins, IgG, in particular, are derived from immune individuals to neutralize pathogens in non-immune recipients [62]. Vaccines based on artificially acquired passive immunity has been approved for infectious disease prophylaxis and therapeutics, especially when vaccines aiming at long-lasting active immunity are not preferred as those diseases are “races against time” [63]; for example, vaccines that have been shown to reduce mortality among patients with severe viral infections such as influenza A viruses and Ebola viruses would involve the intravenous injection of protective antibodies divided from the convalescent plasma of recovered patients or immunized vaccine recipients [64,65]. Likewise, convalescent plasma transfer has been considered as the candidate for immediate treatment for severe COVID-19 patients through measures including direct neutralization and immunomodulation, with the latter involved in (1) the blockage of cytokines or complement, (2) the prevention of DC maturation, or (3) triggering regulatory T cell development [66,67,68].

Active immunity is also transferable after immune cells are trained to induce immunity against specific pathogens ex vivo, thus could be considered as immunotherapy. Immunotherapy, which could be traced back to the late 19th century [69], has emerged as a promising treatment of cancer cells as well as infectious diseases [52,70]. For instance, cellular therapies from donor lymphocyte infusion are utilized to treat cancer relapse following allogeneic hematopoietic stem cell transplantation to bring about graft-vs-leukemia reaction [71,72,73], where antigen-experienced T cells would recognize pathogens such as cytomegalovirus or Epstein–Barr virus. Likewise, antigen-specific T cells acquired by cell expansion or genetically engineered pathogen-specific Tc clones have been applied to infectious diseases [74,75]. In both scenarios, artificial APCs expressing ligands for T cell receptors as well as CD28 co-stimulatory molecules have been developed to prime and expand pathogen-specific effector Tc cells [76]. Moreover, chimeric antigen receptors (CARs) have also been genetically modified in effector cells such as T cells and NK cells, with an extracellular receptor recognizing specific antigens linked plus an intracellular signaling molecule that would activate signal cascades [52]. Conforming to the above principles, clinical trials on COVID-19 patients using APCs and effector lymphocytes including Tc and NK cells have been evaluated for safety and efficacy.

## 4. Challenges and Perspectives

Although elicited active immunity following vaccination provides long-lasting prophylactic immunity against pathogens, how long it takes might exceed the time window for treatment. On the contrary, passive immunity allows for immediate protective immunity by the adoptive transfer of hyperimmunoglobulin derived from convalescent donors. That being said, these non-neutralizing or sub-neutralizing antibodies might bring about either viral infection in target cells expressing Fc receptors, also known as antibody-dependent enhancement (ADE), or immunopathology involving immune cell-mediated cytotoxicity in infected cells that could further induce exaggerated immune reactions, also known as antibody-dependent cellular cytotoxicity (ADCC), both being suggested in previous studies on SARS-CoV-2 [77]. Hence, it requires purification and production of neutralizing antibodies to improve the prognosis of patients with severe COVID-19.

Apart from convalescent donation, direct transfer of cellular immunity could also be achieved through the transfer of ex vivo trained active immunity, also known as immunotherapy. For instance, one trial used engineered ACE2-CAR-NKs to target SARS-CoV-2-infected cells presenting S proteins, and to activate downstream signal transduction, imitating the use of CAR-NKs in cancer immunotherapy [78]. Unlike CAR-T therapy, in which unregulated substantial toxic effects have been clinically observed, activated ACE2-CAR-NKs could be suppressed when attaching to uninfected/healthy cells. Specifically, MHC I molecules expressed by uninfected cells can be recognized by inhibitory receptors of NK cells, followed by inhibitory signal transmission and cytotoxicity alleviation in healthy cells that are facilitated by killer immunoglobulin-like receptors such as KIR2DL and KIR3DL, or C-type lectin receptors including CD94/NKG2A and CD94/NKG2B [79]. Allogenic ACE2-CAR-NK transplantation could thus be an off-the-shelf product for patients with severe COVID-19, although again it takes extensive time and cost.

There are several limitations of this meta-analysis. First, as antibody response or seroconversion rate for each participant were available in phase 2 but not in phase 3 clinical trials, long-term efficacy on the risk of COVID-19 and 28-day efficacy of serum level can not be acquired at the same time through reports on clinical trials of the same phase. Thus, in our study, only seroconversion level but not population efficacy was discussed. Further, although through the 27 reports of clinical trials, we observed the seroconversion and risk of AEs among protein, DNA, RNA, and viral vector vaccines, while delivery systems such as liposome-encapsulated RNA vaccines may improve both antibody response and safety of individual vaccines [80]. As such, future vaccines with optimized delivery may present better safety than that estimated in our meta-analysis. Lastly, due to the limited number of clinical trials reporting on participants with pre-existing chronic diseases, including diabetes mellitus, chronic kidney disease, rheumatic diseases, or participants that were children, we could not determine the safety and seroconversion efficacy of each vaccine on these subgroups.

## 5. Conclusions

**Figure 4:**
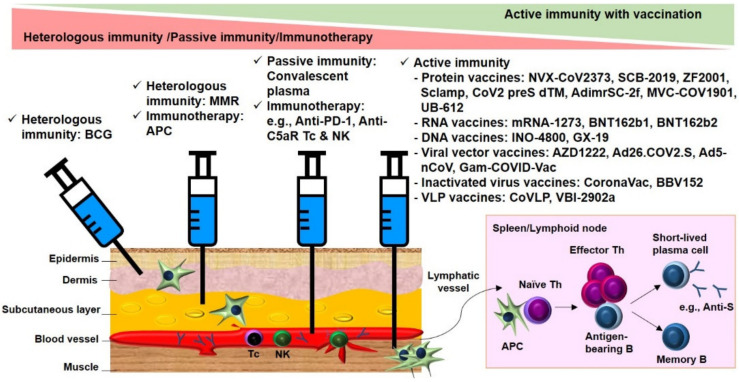
Summary of clinical trials on immune augmentation against SARS-CoV-2 infection. Clinical trials ranged from off-the-shelf BCG or MMR vaccines that aimed at inducing protective heterologous immunity against COVID-19 for healthcare professionals, to direct transfer of hyperimmunoglobulin or ex vivo trained immune cells that aimed at preventing viral dissemination or direct killing of infected cells in COVID-19 patients, then to conventional vaccines with protein vaccines, RNA vaccines, DNA vaccines, viral vector vaccines, inactivated virus vaccines, and VLP vaccines that aimed at COVID-19 prophylaxis via eliciting Th-dependent B memory pathways in healthy adults. BCG, Bacillus Calmette–Guérin; APC, antigen-presenting cell; PD-1: programmed cell death protein-1; Tc, cytotoxic T; NK, natural killer; C5aR, component 5a receptor; VLP, virus-like particle.

In summary, without effective new drugs, immunity manipulation has been considered a promising option to defend against infection. As prophylactic and therapeutic immunity is crucial to fight against SARS-CoV-2 at different stages of disease progression, clinical trials have been launched to evaluate the safety and seroconversion of strategies to manipulate immunity. These trials involve off-the-shelf BCG vaccines for heterologous immunity against SARS-CoV-2 in healthcare providers and direct transfer of immunoglobulin from convalescent donors or ex vivo trained immune cells for preventing viral dissemination or eliminating infected cells in COVID-19 patients, as well as conventional vaccines containing inactivated virus or subunit of pathogens eliciting Th-dependent B memory pathway for specific prophylaxis in healthy adults (Figure 4). Trends toward vaccine-induced active immunity were eminent in clinical trials included for the present systemic review and meta-analysis. The efficacy of humoral immune responses against SARS-CoV-2 for these vaccines was promising, although systemic adverse events were still evident for RNA-based vaccines and viral vector-based vaccines. Further studies are warranted to investigate the underlying mechanisms of effective manipulation of immune responses against COVID-19 with minimized adverse effects.

## 6. Materials and Methods

**Figure 5:**
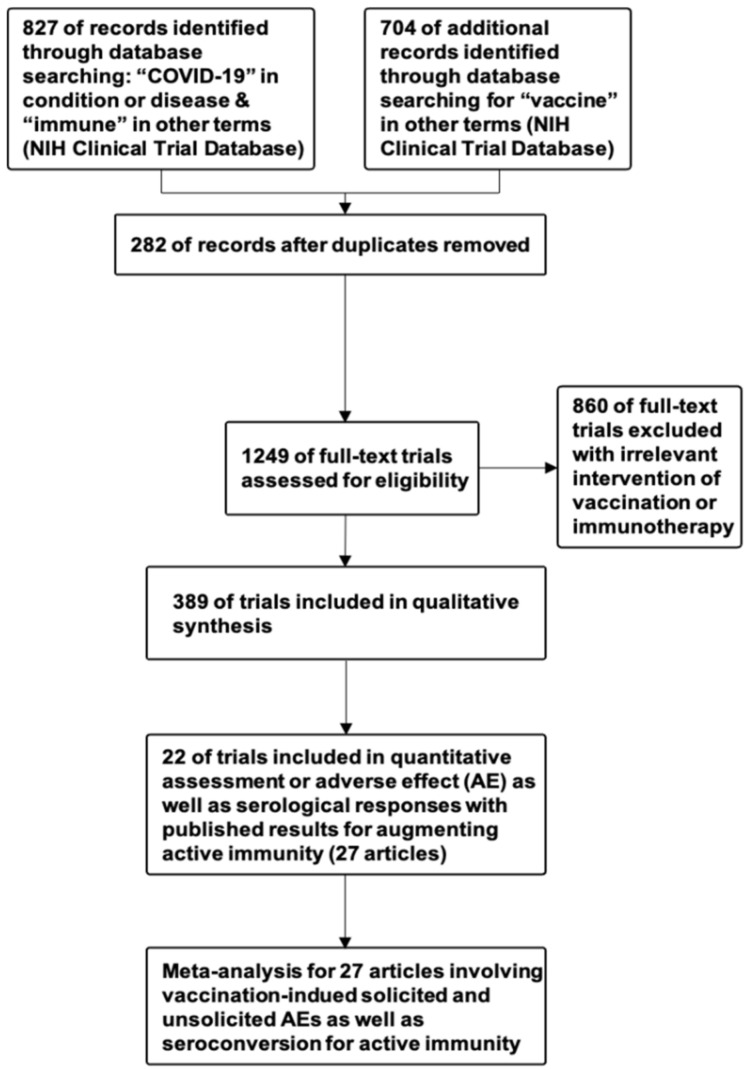
PRISMA flow diagram for selection of clinical trials and published results for meta-analysis.

This study was conducted in accordance with the Preferred Reporting Items for Systematic Review and Meta-analysis of Diagnostic Test Accuracy Studies [81] and Meta-analyses Of Observational Studies in Epidemiology guidelines [82]. Patients or the public were not involved in the design, or conduct, or reporting, or dissemination plans of this research. Inclusion and exclusion criteria are demonstrated in Figure 5.

For the systematic review, we included clinical trials registered on the National Institutes of Health (NIH) Clinical Trial Database (https://clinicaltrials.gov/ accessed on 25 May 2021) that incorporated keywords vaccination and immunity up to 25 May 2021. The search strategy was either “COVID-19” AND “Immune”, or “COVID-19” AND “Vaccine” (Figure 5). To ensure that these trials involve immuno-augmentative mechanisms for developing COVID-19 therapies (Figure 5), four authors (K.S.M., C.C.L., K.J.L, and L.T.W.) screened the trials and identified 389 eligible trials that directly manipulated immunity, including 32 trials that induced training immunity via vaccination, 249 trials that induced active immunity via vaccination, 59 trials that transferred passive immunity, and 59 trials on immunomodulation or enhancement of antiviral immunity based on immunotherapies (Supplementary Table S2).

As for epidemiological data on registered COVID-19 cases in countries with or without Bacillus Calmette–Guérin (BCG) vaccination policy, we estimated the respective COVID-19 mortality rate registered on 12 September at Johns Hopkins Centers for Civic Impact [4] and accordingly evaluated BCG programs among high-income countries listed in the BCG World Atlas [9].

To determine whether, in populations at risk for COVID-19 or patients with COVID-19, there is any difference in antibody response and safety with the four different types of vaccines, including protein vaccines, RNA vaccines, viral vector vaccines, and inactivated vaccines, we performed this systematic review and meta-analysis. In particular, the antibody response was defined as post-vaccination seroconversion levels, and safety was defined as post-vaccination adverse events (AEs), including solicited systemic reactions, solicited local reactions, unsolicited AEs. For the meta-analysis of released results of clinical trials in augmenting active immunity (Figure 5), we searched PubMed, Embase, Scopus, and the Cochrane Central Register of Controlled Trials for articles published through 25 May 2021 that incorporated the trial numbers of included clinical trials registered on the NIH Clinical Trial Database, and identified 27 original articles demonstrating safety and seroconversion of tested trials. The 27 published articles included five for protein-based vaccines [18,19,20,21,22], six for RNA-based vaccines [23,24,25,26,27,28], one for DNA-based vaccine [29], eight for viral vectors [30,31,32,33,34,35,36,37], six for inactivated viruses [38,39,40,41,42,43], and one for virus-like particles (VLPs) [44]. Four authors (K.S.M., C.C.L., K.J.L, and L.T.W.) extracted data on study demographics and both primary and secondary outcomes. The primary outcome was overall safety evinced by post-vaccination AEs in terms of (1) systemic AEs such as fever and fatigue, (2) local reactogenicity or local AEs such as pain and tenderness, and (3) unexpected or unsolicited AEs categorized following the World Health Organization guidance [11,83,84]. The secondary outcome was immunogenicity, as evinced by data on seroconversion.

### Statistical Analysis

Student’s *t*-test was used to compare the differences (mean ± SD) between the intervention and the control group using GraphPad Prism software (CA, USA). A value of *p* < 0.05 was considered statistically significant. Meta-analysis of protein-, RNA-, viral vector-, and inactivated virus-based vaccines were conducted for pooled odds ratios (ORs) with 95% confidence intervals (CIs) with random effect model using *RevMan5 software (Cochrane Collaboration)* [85].

## Figures and Tables

**Table 1:** Distribution of clinical trials on immunity augmentation for COVID-19.

Strategy	Treatment	Total %		Total #		# of Clinical Trial Phases						
						N/A	1	1&2	2	2 & 3	3	4
Heterologous immunity	*Mycobacterium* vaccine	8.0	6.5	32	26	0	0	0	1	0	19	6
	MMR vaccine		0.5		2	0	0	0	0	0	2	0
	Polio vaccine		0.8		3	0	0	0	0	0	1	2
	Zoster Vaccine		0.3		1	0	1	0	0	0	0	0
Active immunity	Protein	62.4	12.3	249	49	0	19	11	10	3	5	1 ^g^
	RNA		17.0		68	15 ^a,b^	8	11	13 ^c,d^	5 ^e^	10	6 ^h^
	DNA		3.3		13	0	4	7	0	2	0	0
	Viral vector		17.5		70	5 ^a^	23	17	6 ^c,d^	3 ^e^	13 ^f^	3 ^g,h,i^
	Bacterial vector		0.3		1	0	1	0	0	0	0	0
	Inactivated		10.5		42	5 ^a,b^	4	8	3	0	14 ^f^	8 ^i^
	Virus-like particle		1.3		5	0	3	1	0	1	0	0
	Live attenuated		0.3		1	0	1	0	0	0	0	0
Passive immunity	Immunoglobulin	14.8	4.5	59	18	0	3	4	3	2	5	1
	Convalescent plasma		10.3		41	8	4	1	19	3	6	0
Immunotherapy	Neutralized antibody/ inhibitor	14.8	7.3	59	30	3	1	1	18	3	3	0
	Cytokine		3.5		14	0	2	1	10	0	1	0
	Immune cell		4.0		16	0	7	9	0	0	0	0
Total # of clinical trial phases					389	33	81	71	81	21	78	24
Total % of clinical trial phases			100.0			8.5	20.8	18.3	20.8	5.4	20.1	6.2

^a, b, c, d, e, f, g, h, i^ Strategies of active immunity are applied to the same clinical trials.

**Table 2:** Distribution of enrolled participants in clinical trials on immunity augmentation for COVID-19.

Strategy	Treatment	Total #		# of Participants in Clinical Trial Phases						
				N/A	1	1&2	2	2 & 3	3	4
Heterologous immunity	*Mycobacterium* vaccine	48,601	41,066	0	0	0	1000	0	29,202	10,864
	MMR vaccine		260	0	0	0		0	260	0
	Polio vaccine		7025	0	0	0	0	0	3600	3425
	Zoster Vaccine		250	0	250	0	0	0	0	0
Active immunity	Protein	878,370	172,672	0	1616	6600	18,016	29,320	117,000	120 ^g^
	RNA		162,052	9502 ^a,b^	981	13,961	6708 ^c,d^	54,550 ^e^	65,500	10,850 ^h^
	DNA		8481	0	298	1105	0	7078	0	0
	Viral vector		271,524	3770 ^a^	2688	9025	3691 ^c,d^	17,930 ^e^	224,000 ^f^	10,420 ^g,h,i^
	Bacterial vector		84	0	84	0	0	0	0	0
	Inactivated/LVP		263,949	2461 ^a,b^	570	5300	1750	30,612	175,790 ^f^	47,466 ^i^
	Live attenuated		48	0	48	0	0	0	0	0
Passive immunity	Immunoglobulin	16,620	2756	0	74	239	226	390	1787	40
	Convalescent plasma		13,864	6424	150	15	2527	688	4060	0
Immunotherapy	Neutralized antibody/ inhibitor	6988	3547	204	50	18	2409	320	546	0
	Cytokine		2329	0	82	80	1129	0	1038	0
	Immune cell		1112	0	280	832	0	0	0	0
Total participants in clinical trial phases			929,359	22,761	7171	37,175	34,816	136,888	618,783	72,765

Participant # in the same clinical trials: ^a^ 100, ^b^ 400, ^c^ 900, ^d^ 1300, ^e^ 4000, ^f^ 4000, ^g^ 120, ^h^ 10,000, ^i^ 300.

**Table 3:** Characteristics of included studies.

Type	Authors (Journal & Year)	NCT Number	Phase	Participants	Vaccination Procedures	Outcome Measures on Safety and Immunogenicity	Storage
Protein	Keech et al. (N Engl J Med 2020) [18]	04368988	1	131	Intramuscular injections of NVX-CoV2373 (5, 25 μg) at day 0 or/and 21	Local, systemic, and unsolicited AEs; anti-S antibody; SARS-CoV-2-neutralizing antibody	2–8 °C
	Richmond et al. (Lancet 2021) [19]	04405908	1	151	Intramuscular injections of SCB-2019 (3, 9, or 30 μg) at days 0 and 21	Local and systemic AEs; anti-SCB-2019 antibody; SARS-CoV-2-neutralizing antibody	2–8 °C
	Yang et al. (Lancet Infect Dis 2021) [20]	04445194 & 04466085	1 & 2	950	Intramuscular injections of ZF2001 at day 0, 30, 60 for phase 1 trial, and at day 0, 30 or day 0, 30, 60 for phase 2 trial	Local, systemic, and unsolicited AEs; anti-RBD antibody; SARS-CoV-2-neutralizing antibody	2–8 °C
	Chappell (Lancet Infect Dis 2021) [21]	04495933	1	120	Intramuscular injections of S-clamp vaccine (5, 15, or 45 μg) at days 0 and 28	Local, systemic, and unsolicited AEs; Anti-clamp antibody; SARS-CoV-2-neutralizing antibody	2–8 °C
	Goepfert et al. (Lancet Infect Dis 2021) [22]	04537208	1 & 2	441	Intramuscular injections of CoV2 preS dTM (1.3 or 2.6 μg) at day 1 for one dose or day 1 and 28 for two doses	Local, systemic, and unsolicited AEs; SARS-CoV-2-neutralizing antibody	2–8 °C
RNA	Anderson et al. (N Engl J Med 2020) [23]	04283461	1	40	Intramuscular injections of mRNA-1273 (25 or 100 μg) at days 1 and 29	Local and systemic AEs; anti-S-2P antibody; anti-RBD antibody; SARS-CoV-2 neutralizing antibody	−20 °C or 2–8 °C
	Chu et al. (Vaccine 2021) [24]	04405076	2	600	Intramuscular injections of mRNA-1273 (50 or 100 μg) at days 1 and 29	Local and systemic AEs; Anti-S antibody; SARS-CoV-2-neutralizing antibody	−20 °C or 2–8 °C
	Jackson et al. (N Engl J Med 2020) [25]	04283461	2	45	Intramuscular injections of mRNA-1273 (25, 100, 250 μg) at days 1 and 29	Local, systemic, and unsolicited AEs; anti-S-2P antibody; anti-RBD antibody; SARS-CoV-2-neutralizing antibody	−20 °C or 2–8 °C
	Mulligan et al. (Nature 2020) [26]	04368728	1 & 2	45	Intramuscular injection of BNT162b1 (10, 30 μg) at day 0 and 21 or BNT162b1 (100 μg) at day0	Local and systemic AEs; anti-RBD antibody; SARS-CoV-2-neutralizing antibody	−80 °C
	Sahin et al. (Nature 2020) [27]	04368728	1 & 2	60	Intramuscular injections of BNT162b1 (1, 10, 30, 50 μg) at day 1 and 22 or BNT162b1 (60 μg) at day 1	Local and systemic AEs; anti-RBD antibody; SARS-CoV-2-neutralizing antibody	−80 °C
	Walsh et al. (N Engl J Med 2020) [28]	04368728	1 & 2	195	Intramuscular injections of BNT162b1 or BNT162b2 (10, 20, 30 μg) at day 0 and 21	Local and systemic AEs; anti-S1 antibody; SARS-CoV-2-neutralizing antibody	−80 °C
DNA	Tebas et al. (EClinicalMedicine 2021) [29]	04336410	1	40	Intrdermal injections of INO-4800 (1, 2 mg) at weeks 0 and 4	Local and systemic AEs; anti-S antibody; SARS-CoV-2-neutralizing antibody	2–8 °C
Viral vector	Zhu et al. (Lancet 2020a) [30]	04313127	1	108	Intramuscular injection of adenovirus type-5 vectored COVID-19 vaccine (5 × 10^10^, 1 × 10^11^, and 1.5 × 10^11^ viral particles) at day 0	Local and systemic AEs; anti-RBD antibody; SARS-CoV-2 neutralizing antibody	N/A
	Folegatti et al. (Lancet 2020) [31]	04324606	1 & 2	1077	Intramuscular injections of AZD1222 (5 × 10^10^ viral particles) at days 0 and 28	Local, systemic, and unsolicited AEs; anti-Spike antibody; SARS-CoV-2 neutralizing antibody	−80 °C or 2–8 K22
	Barrett et al. (Nat Med 2021) [32]	04324606	1 & 2	52	Intramuscular injections of AZD1222 (5 × 10^9^ or 2.5 × 10^10^ viral particles) at days 0 and 28	Local and systemic AEs; anti-S antibody; anti-RBD antibody; SARS-CoV-2-neutralizing antibody	−80 °C or 2–8 °C
	Zhu et al. (Lancet 2020b) [33]	04341389	2	508	Intramuscular injection of adenovirus type-5 vectored COVID-19 vaccine (5 × 10^10^ or 1 × 10^11^ viral particles) at day 0	Local, systemic, and unsolicited AEs; anti-RBD antibody; SARS-CoV-2-neutralizing antibody	N/A
	Ramasamy et al. (Lancet 2021) [34]	04400838	2 & 3	560	Intramuscular injections of AZD1222 (2.2 × 10^10^ or 3.5–6.5 × 10^10^ viral particles) at days 0 and 28	Local and systemic AEs; anti-S antibody; anti-RBD antibody; SARS-CoV-2-neutralizing antibody	−80 °C or 2–8 °C
	Sadoff et al. (N Engl J Med 2021) [35]	04436276	1 & 2	805	Intramuscular injections of Ad26.COV2.S (5 × 10^10^ or 1 × 10^11^ viral particles) at day 1 or/and day 57	Local, systemic, and severe unsolicited AEs; Anti-S antibody; SARS-CoV-2-neutralizing antibody	2–8 °C
	Stephenson et al. (JAMA 2021) [36]	04436276	1	25	Intramuscular injections of Ad26.COV2.S (5 × 10^10^ or 1 × 10^11^ viral particles) at day 1 or/and day 57	Local, systemic, and unsolicited AEs; Anti-S antibody; Anti-RBD antibody SARS-CoV-2-neutralizing antibody	2–8 °C
	Logunov et al. (Lancet 2020) [37]	04436471 & 04437875	1 & 2	76	Intramuscular injections of rAd26-S and rAd5-S at day 0 for phase 1 trial, and at day 0 and 21 for phase 2 trial	Local and systemic AEs; anti-RBD antibody; SARS-CoV-2-neutralizing antibody	Frozen: −18 °C & lyophilized: 2–8 °C
Inactivated virus	Zhang et al. (Lancet Infect Dis 2021) [38]	04352608	1 & 2	744	Intramuscular injections of CoronaVac (3 or 6 μg) at day 0 and 14 or 28 for phase 1or phase 2	Local, systemic, and unsolicited AEs; Anti-RBD antibody; SARS-CoV-2-neutralizing antibody	2–8 °C
	Wu et al. (Lancet Infect Dis 2021) [39]	04383574	1 & 2	422	Intramuscular injections of inactivated CN02 strain at day 0 and 28 for phase 1 trial (3, 6 μg), and at day 0 for phase 2 trial (1.5, 3, 6 μg)	Local and systemic AEs; SARS-CoV-2 neutralizing antibody	2–8 °C
	Che et al. (Clin Infect Dis 2020) [40]	04412538	2	750	Injections of inactivated virus (100 EU or 150 EU viral antigen) at day 0 and boost at day 14 or 28	Local, systemic, and unsolicited AEs; anti-SARS-CoV-2 antibody; SARS-CoV-2-neutralizing antibody	N/A
	Pu et al. (Vaccine 2021) [41]	04412538	1	192	Intramuscular injections of inactivated virus with a D614G mutation in the S protein (50, 100, or 150 EU) at days 0 and 14 or 28.	Local, systemic, and unsolicited AEs; Anti-S antibody; SARS-CoV-2-neutralizing antibody	N/A
	Ella et al. (Lancet Infect Dis 2021a) [42]	04471519	1	375	Intramuscular injections of BBV152 (3 or 6 μg) at days 0 and 14	Local and systemic AEs; anti-S antibody; anti-RBD antibody; SARS-CoV-2 neutralizing antibody	2–8 °C
	Ella et al. (Lancet Infect Dis 2021b) [43]	04471519	2	380	Intramuscular injections of BBV152 (3 or 6 μg) at days 0 and 28	Local and systemic AEs; anti-S antibody; anti-RBD antibody; SARS-CoV-2-neutralizing antibody	2–8 °C
Virus-like particle	Ward et al. (Nat Med 2021) [44]	04450004	1	180	Intramuscular injections of CoVLP (3.75, 7.5, or 15 μg) at days 0 and 21	Local, systemic, and unsolicited AEs; Anti-S antibody; SARS-CoV-2-neutralizing antibody	2–8 °C

## Data Availability

Not applicable.

## Supplementary Materials

The following are available online at https://www.mdpi.com/article/10.3390/pathogens10121537/s1, Table S1: Epidemiological data on COVID-19 cases and CG programs among high income countries, Table S2: Clinical trials for immune augmentation against SARS-CoV-2 virus infection, Figure S1: Epide-miological analyses revealed comparatively low mortality for COVID-19 in high-income countries with BCG vaccination policies; Figure S2: Forest plots and summary estimates for safety of vaccines, defined as the inverse of local adverse events (AEs); Figure S3: Forest plots and summary estimates for safety of vaccines, defined as the inverse of unsolicited AEs.
