# Supplementary material for: Safety and Seroconversion of Immunotherapies against SARS-CoV-2 Infection: A Systematic Review and Meta-Analysis of Clinical Trials
Source: Pathogens. 2021 Nov 24;10(12):1537. doi: 10.3390/pathogens10121537 (PMC8706687; doi:10.3390/pathogens10121537)
Supplement: Supplementary file 1 [file pathogens-10-01537-s001.zip › pathogens-1436034 final supplementary/supplementary figures V3.pdf]

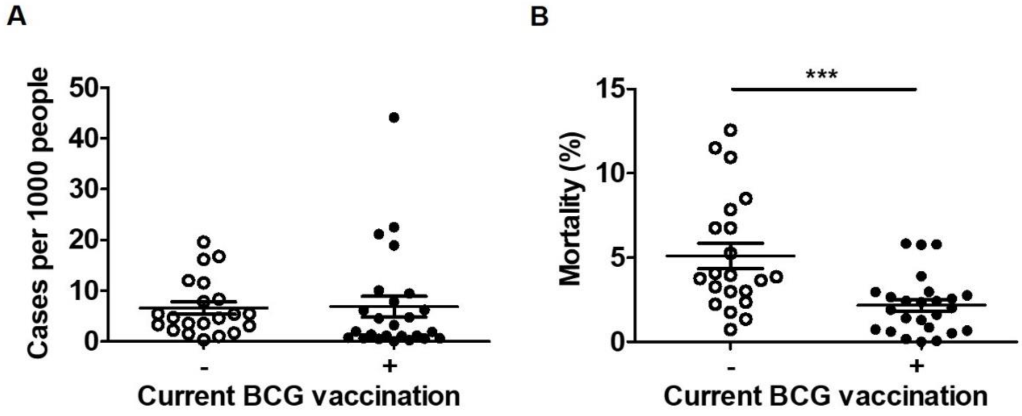

Figure S1. Epidemiological analyses revealed comparatively low mortality for COVID-19 in high-income countries with BCG vaccination policies. (**A**) Incidence rates and (**B**) Mortality rates of reported COVID-19 cases by September 12th, 2020 among high-income countries, whose claimed healthcare data on the BCG World Atlas were considered reflective of the larger population, was summarized.

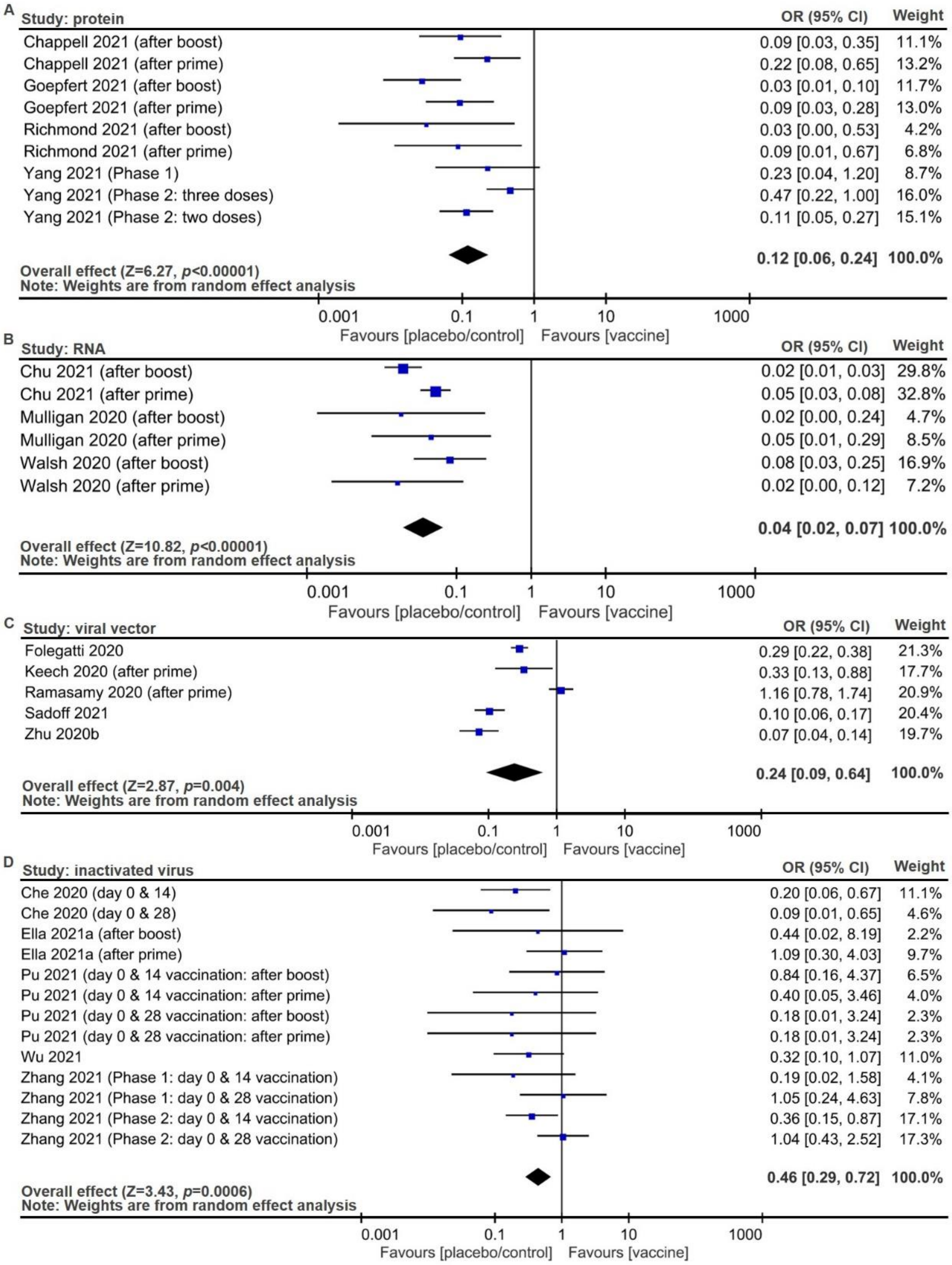

Figure S2. Forest plots and summary estimates for safety of vaccines, defined as the inverse of local adverse events (AEs). Numbers of total participants and vaccinated populations with AEs for (**A**) protein vaccines, (**B**) RNA vaccines, (**C**) viral vector vaccines, and (**D**) inactivated vaccines. Random effect model was used to derive pooled inverse odds ratios (ORs) with 95% Confidence Intervals (CIs).

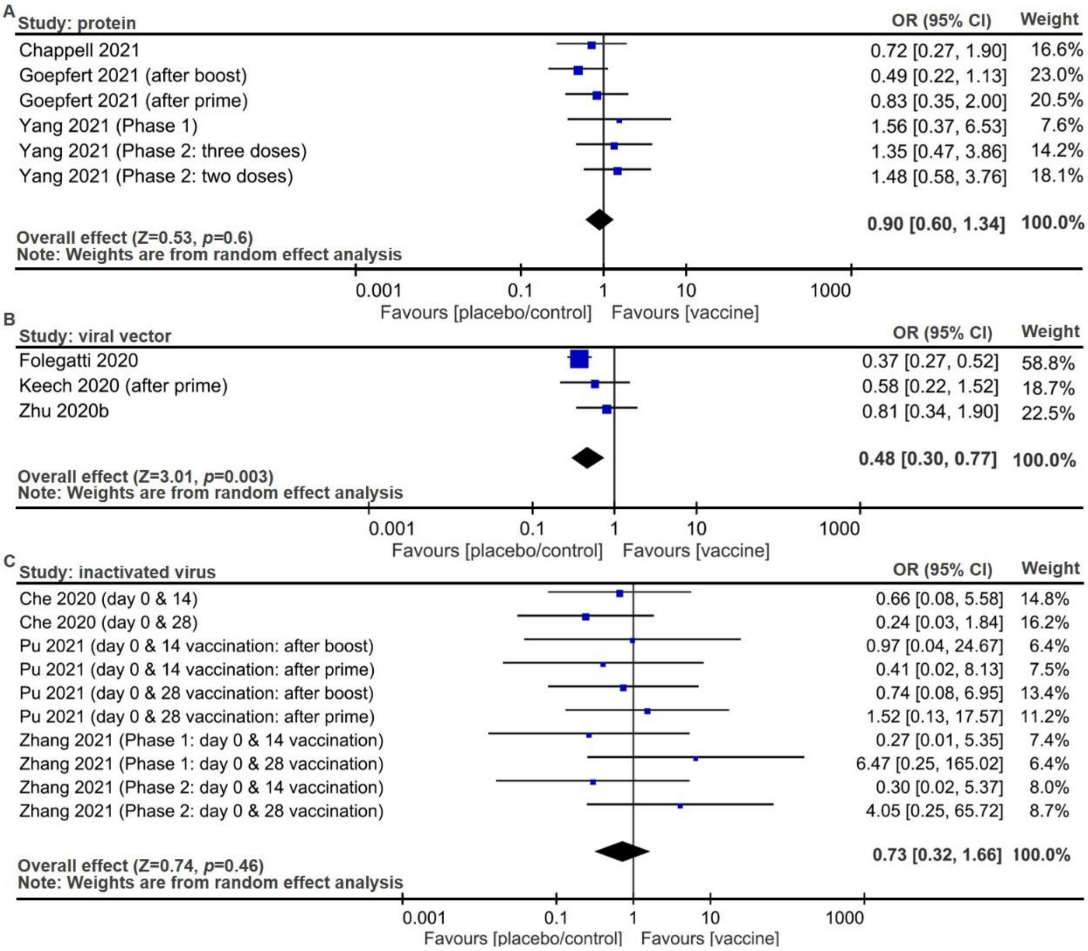

Figure S3. Forest plots and summary estimates for safety of vaccines, defined as the inverse of unsolicited AEs. Numbers of total participants and vaccinated populations with AEs for (**A**) protein vaccines, (**B**) viral vector vaccines, and (**C**) inactivated vaccines. Random effect model was used to derive the inverse ORs with 95% CIs.
