# Supplementary material for: Safety and Seroconversion of Immunotherapies against SARS-CoV-2 Infection: A Systematic Review and Meta-Analysis of Clinical Trials
Source: Pathogens. 2021 Nov 24;10(12):1537. doi: 10.3390/pathogens10121537 (PMC8706687; doi:10.3390/pathogens10121537)
Supplement: Supplementary file 1 [file pathogens-10-01537-s001.zip › pathogens-1436034 final supplementary/Supplementary table 1.pdf]

Table S1. Epidemiological data on COVID-19 cases and BCG programs among high income countries

Location	Confirmed	Cases per 1M people	Deaths	mortality	Current BCG vaccination
Italy	284,796	4,727	35,767	12.56	no
United Kingdom	361,677	5,444	41,614	11.51	no
Belgium	90,568	7,859	9,917	10.95	no
France	363,350	5,417	30,893	8.50	no
Netherlands	79,781	4,572	6,252	7.84	no
Sweden	86,505	8,371	5,846	6.76	no
Canada	135,626	3,571	9,163	6.76	no
Spain	566,326	12,024	29,747	5.25	no
Andorra	1,301	16,778	53	4.07	no
Finland	8,512	1,540	336	3.95	no
Slovenia	3,498	1,670	135	3.86	no
Switzerland	46,239	5,385	1,740	3.76	no
Germany	258,519	3,109	9,421	3.64	no
Denmark	19,216	3,300	629	3.27	no
Australia	26,565	1,035	797	3.00	no
United States	6,462,503	19,610	192,795	2.98	no
Austria	31,827	3,575	750	2.36	no
Norway	11,867	2,211	265	2.23	no
Luxembourg	7,088	11,546	124	1.75	no
New Zealand	1,795	361	24	1.34	no
Israel	148,564	16,183	1,090	0.73	no
Ireland	30,571	6,212	1,781	5.83	yes
Hungary	10,909	1,116	631	5.78	yes
Iran	397,801	4,774	22,913	5.76	yes
Barbados	180	627	7	3.89	yes
Poland	73,047	1,903	2,169	2.97	yes
Portugal	62,813	6,112	1,855	2.95	yes
Chile	430,535	22,533	11,850	2.75	yes
Lithuania	3,243	1,161	86	2.65	yes
Uruguay	1,759	500	45	2.56	yes
Estonia	2,632	1,981	64	2.43	yes
Latvia	1,448	759	35	2.42	yes
Greece	12,734	1,187	300	2.36	yes
Hong Kong	4,926	657	99	2.01	yes
Japan	73,901	587	1,412	1.91	yes
Croatia	13,107	3,215	211	1.61	yes
Taiwan	498	21	7	1.41	yes
Saudi Arabia	324,407	9,481	4,213	1.30	yes
Oman	88,337	18,937	762	0.86	yes
Seychelles	137	1,403	1	0.73	yes
Malta	2,247	4,553	15	0.67	yes
Kuwait	93,475	21,148	557	0.60	yes
United Arab Emira	77,842	7,870	398	0.51	yes
Qatar	121,287	44,148	205	0.17	yes
Singapore	57,315	10,049	27	0.05	yes
Greenland	14	250	0	0.00	yes
