# Supplementary material for: Safety and Seroconversion of Immunotherapies against SARS-CoV-2 Infection: A Systematic Review and Meta-Analysis of Clinical Trials
Source: Pathogens. 2021 Nov 24;10(12):1537. doi: 10.3390/pathogens10121537 (PMC8706687; doi:10.3390/pathogens10121537)
Supplement: Supplementary file 1 [file pathogens-10-01537-s001.zip › pathogens-1436034 final supplementary/Supplementary table 2.pdf]

Table S2. Clinical trials for immune augmentation against SARS-CoV-2 virus infection

Strategy		NCT Number	Title	Route	Treatment	Age	Phases	Enrollment	Start Date
		NCT04328441	Reducing Health Care Workers Absenteeism in Covid-19 Pandemic Through BCG Vaccine	IC	BCG	18 Years and older	3	1500	2020-03-25
		NCT04327206	BCG Vaccination to Protect Healthcare Workers Against COVID-19	ID	BCG	18 Years and older	3	4170	2020-03-30
		NCT04417335	Reducing COVID-19 Related Hospital Admission in Elderly by BCG Vaccination	IC	BCG	60 Years and older	4	2014	2020-04-16
		NCT04350931	Application of BCG Vaccine for Immune-prophylaxis Among Egyptian Healthcare Workers During the Pandemic of COVID-19	ID	BCG	18 Years and older	3	900	2020-04-20
		NCT04362124	Performance Evaluation of BCG vs COVID-19	ID	BCG	18 Years to 65 Years	3	1000	2020-04-20
		NCT04348370	BCG Vaccine for Health Care Workers as Defense Against SARS-COV2	ID	BCG	18 Years to 74 Years	4	700	2020-04-20
		NCT04373291	Using BCG Vaccine to Protect Health Care Workers in the COVID-19 Pandemic	ID	BCG	18 Years to 100 Years	3	1500	2020-05-01
		NCT04379336	BCG Vaccination for Healthcare Workers in COVID-19 Pandemic	ID	BCG	18 Years and older	3	500	2020-05-04
		NCT04384549	Efficacy of BCG Vaccination in the Prevention of COVID19 Via the Strengthening of Innate Immunity in Health Care Workers	ID	BCG	18 Years and older	3	1120	2020-05-11
		NCT04387409	A Phase III, Double-blind, Randomized, Placebo-controlled Multicentre Clinical Trial to Assess the Efficacy and Safety of VPM1002 in Reducing Healthcare Professionals' Absenteeism in the SARS-CoV-2 Pandemic by Modulating the Immune System	ID	VPM1002	18 Years and older	3	1200	2020-05-18
		NCT04387409	Study to Assess VPM1002 in Reducing Healthcare Professionals' Absenteeism in COVID-19 Pandemic	ID	VPM1002	18 Years and older	3	1200	2020-05-25
		NCT04414267	Bacillus Calmette-guéri Vaccination to Prevent COVID-19	ID	BCG	50 Years and older	4	900	2020-05-26
		NCT04369794	COVID-19: BCG As Therapeutic Vaccine, Transmission Limitation, and Immunoglobulin Enhancement	ID	BCG	18 Years and older	4	1000	2020-06-01
	Mycobacterium vaccine	NCT04435379	Study to Assess VPM1002 in Reducing Hospital Admissions and/or Severe Respiratory Infectious Diseases in Elderly in COVID-19 Pandemic	ID	VPM1002	60 Years and older	3	2038	2020-06-01
		NCT04439045	Efficacy and Safety of VPM1002 in Reducing SARS-CoV-2 (COVID-19) Infection Rate and Severity	ID	VPM1002	18 Years and older	3	3626	2020-06-14
Training immunity		NCT04442048	Immunization With IMM-101 vs Observation for Prevention of Respiratory and Severe COVID-19 Related Infections in Cancer Patients at Increased Risk of Exposure	UNS	IMM-101	18 Years and older	3	1500	2020-07-01
		NCT04475302	BCG Vaccine in Reducing Morbidity and Mortality in Elderly Individuals in COVID-19 Hotspots	ID	BCG	60 Years to 80 Year	3	2175	2020-07-01
		NCT04461379	Prevention, Efficacy and Safety of BCG Vaccine in COVID-19 Among Healthcare Workers	ID	BCG	18 Years and older	3	908	2020-07-06
		NCT04648800	Clinical Trial Evaluating the Effect of BCG Vaccination on the Incidence and Severity of SARS-CoV-2 Infections Among Healthcare Professionals During the COVID-19 Pandemic in Poland	ID	BCG	25 Years and older	3	1000	2020-07-07
		NCT04453488	Clinical Trial to Evaluate the Efficacy of RUTII® Against SARS-COV-2 Infection (COVID-19) in Healthcare Workers	SC	RUTI®	18 Years and older	3	315	2020-07-30
		NCT04537663	Prevention Of Respiratory Tract Infection And Covid-19 Through BCG Vaccination In Vulnerable Older Adults	ID	BCG	60 Years and older	4	5200	2020-08-25
		NCT04534803	BCG Against Covid-19 for Prevention and Amelioration of Severity Trial (BAC to the PAST)	ID	BCG	70 Years and older	3	2100	2020-09-01
		NCT04542330	Using BCG to Protect Senior Citizens During the COVID-19 Pandemic	ID	BCG	65 Years to 110 Years	3	1900	2020-09-15

	NCT04659941	Use of BCG Vaccine as a Preventive Measure for COVID-19 in Health Care Workers	ID	BCG	18 Years and older	2	1000	2020-10-01
	NCT04641858	BCG to Reduce Absenteeism Among Health Care Workers During the COVID-19 Pandemic	ID	BCG	18 Years and older	4	1050	2020-11-01
	NCT04632537	BCG Vaccination to Prevent COVID-19	ID	BCG	18 Years to 64 Years	3	550	2020-11-01
MMR vaccine	NCT04357028	Measles Vaccine in HCW	SC	MMR	18 Years to 50 Years	3	200	2020-05-01
	NCT04475081	Use of a Live Attenuated Vaccine as an Immune-based Preventive Against COVID-19-associated Sepsis	SC	MMR	18 Years to 70 Years	3	60	2020-08-17
Polio vaccine	NCT04445428	OPV as Potential Protection Against COVID-19	ORAL	OPV	50 Years and older	4	3400	2020-07-15
	NCT04540185	A Phase 3 Randomized Double Blind Efficacy and Safety Study of Oral Polio Vaccine and NA-831 for Covid-19	ORAL	OPV	18 Years and older	3	3600	2020-11-01
	NCT04639375	Polio Vaccine (IPV) for SARS-CoV-2 and Prevention of Coronavirus Disease (COVID-19)	UNS	IPV	18 Years to 80 Years	4	25	2020-11-15
Zoster vaccine	NCT04523246	Training the Innate Immune System Against SARS-CoV-2 (COVID-19) Using the Shingrix Vaccine in Nursing Home Residents	IM	Zoster	65 Years to 100 Years	1	250	2020-09-01
	NCT04833101	Study on Sequential Immunization of Recombinant COVID-19 Vaccine (Ad5 Vector) and RBD-based Protein Subunit Vaccine	UNS	ZF2001	18 Years and older	4	120	2021-04-07
	NCT04368988	Evaluation of the Safety and Immunogenicity of a SARS-CoV-2 rS (COVID-19) Nanoparticle Vaccine With/Without Matrix-M Adjuvant	IM	SARS-CoV-2 rS	18 Years to 59 Years	1	131	2020-05-15
	NCT04405908	SCB-2019 as COVID-19 Vaccine	IM	SCB-2019	18 Years to 75 Years	1	150	2020-06-20
	NCT04445194	Phase I Clinical Study of Recombinant Novel Coronavirus Vaccine	IM	SARS-CoV-2 rS (CHO cell)	18 Years to 59 Year	1	50	2020-06-22
	NCT04453852	Monovalent Recombinant COVID19 Vaccine	UNS	SARS-CoV-2 rS	18 Years to 65 Years	1	40	2020-06-30
	NCT04428073	Therapeutic Vaccine Trial of COVID-19 for Severe Acute Respiratory Syndrome Coronavirus 2 (SARS-CoV-2) Infection	UNS	Advax-CpG55.2	18 Years to 60 Years	1	32	2020-07-01
	NCT04466085	Clinical Study of Recombinant Novel Coronavirus Vaccine	IM	SARS-CoV-2 rS (CHO cell)	18 Years to 59 Years	2	900	2020-07-12
	NCT04495933	A Study on the Safety, Tolerability and Immune Response of SARS-CoV-2 Sclamp (COVID-19) Vaccine in Healthy Adults	IM	Sclamp	18 Years to 55 Years	1	120	2020-07-13
	NCT03305341	Discovery Stage (Proof-of-concept) COVID-19 Antigen Presentation Therapeutic Vaccine	ID	SARS-CoV-2 rS/GM-CSF	22 Years to 72 Years	1	20	2020-07-18
	NCT04527575	Study of the Safety, Reactogenicity and Immunogenicity of "EpiVacCorona" Vaccine for the Prevention of COVID-19	IM	EpiVacCorona	18 Years to 60 Years	1 & 2	100	2020-07-27
	NCT04533399	A Study Looking at the Effectiveness and Safety of a COVID-19 Vaccine in South African Adults	IM	SARS-CoV-2 rS/Matrix-M1	18 Years to 64 Years	2	2904	2020-08-17
	NCT04550351	Recombinant New Coronavirus Vaccine (CHO Cells) to Prevent SARS-CoV-2 Phase I Clinical Trial (??0 Years Old)	IM	SARS-CoV-2 rS (CHO cell)	60 Years and older	1 & 2	50	2020-08-19
	NCT04522089	A Study to Evaluate the Safety and Immunogenicity of COVID-19 (AdimrSC-2f) Vaccine	UNS	AdimrSC-2f	20 Years to 60 Years	1	70	2020-08-20
	NCT04530656	Phase I Trial of a Recombinant SARS-CoV-2 Vaccine (Sf9 Cell)	IM	SARS-CoV-2 rS (Sf9 Cell)	18 Years and older	1	168	2020-08-28
	NCT04487210	A Phase I, Prospective, Open-Labeled Study to Evaluate the Safety and Immunogenicity of MVC-COV1901	IM	MVC-COV1901	20 Years to 50 Year	1	45	2020-09-01
	NCT04537208	Study of Recombinant Protein Vaccine Formulations Against COVID-19 in Healthy Adults 18 Years of Age and Older	IM	SARS-CoV-2 rS	18 Years and older	1 & 2	440	2020-09-03
	NCT04473690	KBP-201 COVID-19 Vaccine Trial in Healthy Volunteers	UNS	KBP-COVID-19	18 Years to 70 Years	1 & 2	180	2020-09-14
	NCT04545749	A Study to Evaluate the Safety, Tolerability, and Immunogenicity of UB-612 COVID-19 Vaccine	UNS	UB-612	20 Years to 55 Years	1	60	2020-09-25

	NCT04583995	A Study Looking at the Effectiveness, Immune Response, and Safety of a COVID-19 Vaccine in Adults in the United Kingdom	IM	SARS-CoV-2 rS/Matrix M1	18 Years to 84 Years	3	15000	2020-09-28
	NCT04546841	Safety and Immunogenicity Trial of Multi-peptide Vaccination to Prevent COVID-19 Infection in Adults	SC	SARS-CoV-2 rS	18 Years and older	1	36	2020-09-30
	NCT04636333	Phase I Trial of a Recombinant SARS-CoV-2 Vaccine (CHO Cell)	IM	SARS-CoV-2 rS (CHO cell)	18 Years and older	1	216	2020-10-30
	NCT04611802	A Study Looking at the Efficacy, Immune Response, and Safety of a COVID-19 Vaccine in Adults at Risk for SARS-CoV-2	IM	SARS-CoV-2 rS/Matrix-M1	18 Years and older	3	30000	2020-11-01
	NCT04640402	A Phase 2 Clinical Trial of Recombinant Corona Virus Disease-19 (COVID-19) Vaccine (Sf9 Cells)	UNS	SARS-CoV-2 rS (Sf9 cells)	18 Years to 85 Years	2	960	2020-11-17
	NCT04780035	Study of the Tolerability, Safety, Immunogenicity and Preventive Efficacy of the EpiVacCorona Vaccine for the Prevention of COVID-19	IM	EpiVacCorona	18 Years and older	3	3000	2020-11-18
	NCT04683484	A Clinical Trial to Assess the Safety and Immunogenicity of Nanocovax in Heathy Volunteers	IM	Nanocovax	12 Years to 75 Years	1 & 2	620	2020-12-10
Protein vaciine	NCT04806529	An Efficacy, Immunogenicity and Safety Study Investigating an Adjuvanted SARS-CoV-2 Influenza Vaccine to Protect Against COVID-19 in Adults Over Aged 18 Years-old and Older	IM	aCoV2	18 Years and older	2 & 3	0	2020-12-15
	NCT04646590	A Phase III Clinical Trial to Determine the Safety and Efficacy of ZF2001 for Prevention of COVID-19	IM	SARS-CoV-2 rS (CHO cell)	18 Years and older	3	29000	2020-12-16
	NCT04760743	Safety and Immunogenicity of a SARS-CoV-2 Vaccine (NBP2001) in Healthy Adults (COVID-19)	IM	NBP2001	19 Years to 55 Years	1	50	2020-12-17
	NCT04695652	A Study to Evaluate MVC-COV1901 Vaccine Against COVID-19 in Adult	IM	MVC-COV1901	20 Years and older	2	3700	2020-12-30
	NCT04742738	Safety and Immunogenicity Study of SARS-CoV-2 Nanoparticle Vaccine (GBP510) Adjuvanted With Aluminum Hydroxide (COVID-19)	IM	GBP510	19 Years to 85 Years	1 & 2	260	2021-01-20
	NCT04773067	A Study to Evaluate UB-612 COVID-19 Vaccine in Adolescent, Younger and Elderly Adult Volunteers	UNS	UB-612	12 Years to 85 Years	2	3850	2021-01-30
	NCT04683224	A Study to Evaluate the Safety, Immunogenicity, and Efficacy of UB-612 COVID-19 Vaccine	IM	UB-612	18 Years and older	2 & 3	7320	2021-02-01
	NCT04718467	Phase IIb Clinical Trial of Recombinant Novel Coronavirus Pneumonia (COVID-19) Vaccine (Sf9 Cells)	IM	SARS-CoV-2 rS (Sf9 cells)	18 Years to 85 Years	2	4000	2021-02-01
	NCT04750343	Safety and Immunogenicity Study of SARS-CoV-2 Nanoparticle Vaccine (GBP510) Adjuvanted With or Without AS03 (COVID-19)	IM	GBP510	19 Years to 85 Years	1 & 2	320	2021-02-03
	NCT04686773	Study in Adults to Determine the Safety and Immunogenicity of AZD1222, a Non-replicating ChAdOx1 Vector Vaccine, Given in Combination With rAd26-S, Recombinant Adenovirus Type 26 Component of Gam-COVID-Vac Vaccine, for the Prevention of COVID-19.	IM	AZD1222	18 Years to 100 Years	2	100	2021-02-10
	NCT04702178	A Clinical Trial of COVAC-2 in Healthy Adults	IM	COVAC-2	18 Years and older	1	108	2021-02-10
	NCT04758962	A Study of the Safety of and Immune Response to Varying Doses of a Vaccine Against COVID-19 in Healthy Adults	IM	CoV2 SAM	18 Years to 50 Year	1	40	2021-02-15
	NCT04783311	Safety, Tolerance and Immunogenicity of EuCorVac-19 for the Prevention of COVID-19 in Healthy Adults	IM	EuCorVac-19	19 Years to 75 Years	1 & 2	280	2021-02-23
	NCT04712110	A Study of TAK-019 in Healthy Japanese Adults (COVID-19)	IM	TAK-019	20 Years and older	1 & 2	200	2021-02-24
	NCT04762680	Study of Recombinant Protein Vaccine With Adjuvant Against COVID-19 in Adults 18 Years of Age and Older	IM	VAT00002	18 Years and older	2	722	2021-02-24

NCT04672395	A Controlled Phase 2/3 Study of Adjuvanted Recombinant SARS-CoV-2 Trimeric S-protein Vaccine (SCB-2019) for the Prevention of COVID-19	IM	SCB-2019	18 Years and older	2 & 3	22000	2021-03-01
NCT04813562	Immunogenicity and Safety of Recombinant COVID-19 Vaccine (CHO Cells)	UNS	SARS-CoV-2 rS (CHO cells)	18 Years to 85 Years	2	480	2021-03-23
NCT04818801	Safety, Reactogenicity and Immunogenicity Study of ReCOV	IM	SARS-CoV-2 rS (CHO cells)	18 Years to 80 Years	1	160	2021-04-01
NCT04784767	SARS-COV-2-Spike-Ferritin-Nanoparticle (SpFN) Vaccine With ALFQ Adjuvant for Prevention of COVID-19 in Healthy Adults	UNS	SPFN_1B-06-PL	18 Years to 55 Years	1	72	2021-04-05
NCT04681092	Anti-COVID19 AKS-452 - ACT Study	IM	AKS-452	18 Years to 65 Years	1 & 2	130	2021-04-12
NCT04869592	A Clinical Trial to Evaluate the Recombinant SARS-CoV-2 Vaccine (CHO Cell) for COVID-19	IM	SARS-CoV-2 rS	3 Years and older	1 & 2	3580	2021-04-25
NCT04822025	A Study to Evaluate MVC-COV1901 Vaccine Against COVID-19 in Elderly Adults	IM	MVC-COV1901	65 Years and older	2	400	2021-05-01
NCT04885361	To Evaluate the Safety, and Immunogenicity of Vaccine Candidate Against COVID-19, in Healthy Adults	SC	CoVepiT	18 Years to 45 Years	1	48	2021-05-10
NCT04887207	A Global Phase III Clinical Trial of Recombinant COVID-19 Vaccine (Sf9 Cells)	UNS	SARS-CoV-2 Rs (Sf9 cells)	18 Years and older	3	40000	2021-06-01
NCT04706143	Immunologic Responses to Single and Double Doses of COVID-19 Vaccines in Egyptians	UNS	mRNA vs viral vector vs inactivated	25 Years to 65 Years	N/A	100	2021-01-15
NCT04760132	National Cohort Study of Effectiveness and Safety of SARS-CoV-2/COVID-19 Vaccines (ENFORCE)	UNS	BNT162b2 vs mRNA-1273 vs AZD1222	18 Years and older	4	10000	2021-02-08
NCT04885764	Profiling Antibody Status and Vaccine Effectiveness in Post Vaccination With SARS CoV2 in Ain Shams University	IM	Sinopharm vs AZD1222	18 Years and older	2 & 3	4000	2021-02-23
NCT04776317	Chimpanzee Adenovirus and Self-Amplifying mRNA Prime-Boost Prophylactic Vaccines Against SARS-CoV-2 in Healthy Adults	IM	ChAdV68-S vs SAM-LNP-S	18 Years to 99 Years	1	140	2021-03-25
NCT04800133	Covid-19 Vaccination in Adolescents	IM	BNT162b2 vs mRNA-1273 vs AZD1222	11 Years to 100 Years	2	900	2021-04-01
NCT04894435	Mix and Match of the Second COVID-19 Vaccine Dose for Safety and Immunogenicity	IM	BNT162b2 vs mRNA-1273 vs AZD1222	18 Years to 99 Years	2	1300	2021-05-01
NCT04898946	Serological Response to mRNA and Inactivated COVID-19 Vaccine in Health Care Workers in Hong Kong	UNS	Biological: CoronaVac Vaccine|Biological: BNT162b2 Vaccine	18 Years and older	N/A	400	2021-03-08
NCT04283461	Safety and Immunogenicity Study of 2019-nCoV Vaccine (mRNA-1273) for Prophylaxis SARS CoV-2 Infection (COVID-19)	IM	mRNA-1273	18 Years to 99 Years	2	105	2020-03-03
NCT04380701	A Trial Investigating the Safety and Effects of Four BNT162 Vaccines Against COVID-2019 in Healthy Adults	IM	BNT162	18 Years to 55 Years	1 & 2	200	2020-04-23
NCT04368728	Study to Describe the Safety, Tolerability, Immunogenicity, and Potential Efficacy of RNA Vaccine Candidates Against COVID-19 in Healthy Adults	IM	BNT162	18 Years to 85 Years	1 & 2	7600	2020-04-29
NCT04405076	Dose-Confirmation Study to Evaluate the Safety, Reactogenicity, and Immunogenicity of mRNA-1273 COVID-19 Vaccine in Adults Aged 18 Years and Older	UNS	mRNA-1273	18 Years and older	2	600	2020-05-29
NCT04449276	A Study to Evaluate the Safety, Reactogenicity and Immunogenicity of Vaccine CVnCoV in Healthy Adults	IM	CVnCoV	18 Years to 60 Years	1	168	2020-06-18
NCT04470427	A Study to Evaluate Efficacy, Safety, and Immunogenicity of mRNA-1273 Vaccine in Adults Aged 18 Years and Older to Prevent COVID-19	IM	mRNA-1273	18 Years and older	3	30000	2020-07-27

	NCT04523571	Safety and Immunogenicity of SARS-CoV-2 mRNA Vaccine (BNT162b1) in Chinese Healthy Subjects	IM	BNT162b1	18 Years to 85 Years	1	144	2020-07-28
	NCT04480957	Ascending Dose Study of Investigational SARS-CoV-2 Vaccine ARCT-021 in Healthy Adult Subjects	IM	ARCT-021	21 Years to 80 Years	1 & 2	92	2020-08-04
	NCT04515147	A Dose-Confirmation Study to Evaluate the Safety, Reactogenicity and Immunogenicity of Vaccine CVnCoV in Healthy Adults	IM	CVnCoV 6	18 Years and older	2	691	2020-08-17
	NCT04537949	A Trial Investigating the Safety and Effects of One BNT162 Vaccine Against COVID-19 in Healthy Adults	IM	BNT162b3	18 Years to 85 Years	1 & 2	120	2020-09-09
	NCT04588480	Study to Evaluate the Safety, Tolerability, and Immunogenicity of an RNA Vaccine Candidate Against COVID-19 in Healthy Japanese Adults	IM	BNT162b2	20 Years to 85 Years	1 & 2	160	2020-10-21
	NCT04649021	Safety and Immunogenicity of SARS-CoV-2 mRNA Vaccine (BNT162b2) in Chinese Healthy Population	IM	BNT162b2	18 Years to 85 Years	2	950	2020-12-04
	NCT04649151	A Study to Evaluate the Safety, Reactogenicity, and Effectiveness of mRNA-1273 Vaccine in Adolescents 12 to <18 Years Old to Prevent COVID-19	IM	mRNA-1273	12 Years to 17 Years	2 & 3	3000	2020-12-09
	NCT04652102	A Study to Determine the Safety and Efficacy of SARS-CoV-2 mRNA Vaccine CVnCoV in Adults for COVID-19	IM	CVnCoV	18 Years and older	2 & 3	36500	2020-12-14
	NCT04674189	A Study to Evaluate the Safety and Immunogenicity of Vaccine CVnCoV in Healthy Adults in Germany for COVID-19	IM	CVnCoV	18 Years and older	3	2520	2020-12-23
	NCT04842708	Evaluation of Anti-COVID 19 Pfizer Vaccination Effect on COVID 19 Detection Using Breath Analysis	UNS	BNT162b2	18 Years to 75 Years	N/A	50	2020-12-24
	NCT04566276	ChulaCov19 mRNA Vaccine in Healthy Adults	IM	ChulaCov19	18 Years to 75 Years	1	96	2021-01-01
	NCT04713163	COVID-19 Vaccine Induced Immunity	UNS	BNT162b2 or mRNA-1273	18 Years and older	N/A	200	2021-01-01
	NCT04728347	Open Label Extension Study to Assess the Safety and Long-Term Immunogenicity of ARCT-021	UNS	ARCT-021	21 Years to 80 Years	2	106	2021-01-04
	NCT04668339	A Trial Evaluating the Safety and Effects of an RNA Vaccine ARCT-021 in Healthy Adults	UNS	ARCT-021	18 Years and older	2	600	2021-01-07
	NCT04715438	Vaccination Against COVID-19 in Cancer	UNS	mRNA-1273	18 Years and older	N/A	873	2021-01-08
	NCT04765436	PTX-COVID19-B, an mRNA Humoral Vaccine, is Intended for Prevention of COVID-19 in a General Population. This Study is Designed to Evaluate Safety, Tolerability, and Immunogenicity of PTX-COVID19-B Vaccine in Healthy Seronegative Adults Aged 18-64	IM	PTX-COVID19-B	18 Years to 64 Years	1	60	2021-01-14
	NCT04677660	A Study of TAK-919 in Healthy Japanese Adults (COVID-19)	IM	mRNA-1273	20 Years and older	1 & 2	200	2021-01-21
	NCT04733807	Antibodies Response to mRNA Vaccine Against Covid-19	UNS	BNT162b2	18 Years to 80 Years	N/A	600	2021-01-28
	NCT04748172	COVID-19 Vaccine and Ovarian Reserve	UNS	BNT162b2 or mRNA-1273	18 Years to 42 Years	N/A	200	2021-02-01
	NCT04862806	Safety, Efficacy of BNT162b2 mRNA Vaccine in CLL	UNS	BNT162b2	18 Years and older	N/A	1000	2021-02-01
	NCT04748471	Immunogenecity and Safety of VaccinemRNA-1273 in Elderly Volunteers (Over 65 y) Compared to Younger Ones (18-45y)	UNS	mRNA-1273	18 Years and older	2	180	2021-02-10
	NCT04756817	Immunogenicity of the BNT162b2 Covid-19 Vaccine in Elderly People Aged 85 and Older in Greece	UNS	BNT162b2	85 Years to 100 Years	N/A	500	2021-02-13
RNA vaccine	NCT04713553	A Phase 3 Study to Evaluate the Safety, Tolerability, and Immunogenicity of Multiple Production Lots and Dose Levels of BNT162b2 Against COVID-19 in Healthy Participants	IM	BNT162b2	12 Years to 50 Years	3	1530	2021-02-15

NCT04839315	COVID-19 Vaccination in Rheumatic Disease Patients	UNS	BNT162b2 or mRNA-1273	18 Years and older	1	100	2021-02-15
NCT04754594	Study to Evaluate the Safety, Tolerability, and Immunogenicity of SARS CoV-2 RNA Vaccine Candidate (BNT162b2) Against COVID-19 in Healthy Pregnant Women 18 Years of Age and Older	IM	BNT162b2	Child, Adult, Older Adult	2 & 3	4000	2021-02-16
NCT04811391	COVID-19 Vaccine Effectiveness in Albanian Health Workers	UNS	BNT162b2	16 Years and older	N/A	1500	2021-02-19
NCT04780659	COVID-19 Vaccination of Immunodeficient Persons (COVAXID)	UNS	BNT162b2	18 Years and older	4	540	2021-02-23
NCT04784689	Host Immune Response to Novel RNA COVID-19 Vaccination	UNS	RNA vaccine	18 Years and older	n	400	2021-02-24
NCT04769258	Effectiveness and Safety of COVID-19 Vaccine in Patients With IBD Treated With Immunomodulatory or Biological Drugs (ESCAPE-IBD)	UNS	BNT162b2	18 Years and older	N/A	1380	2021-03-01
NCT04824638	BNT162b2 Vaccination With Two Doses in COVID-19 Negative Adult Volunteers and With a Single Dose in COVID-19 Positive Adult Volunteers	IM	BNT162b2	18 Years and older	2	300	2021-03-08
NCT04813796	A Study to Evaluate Safety, Reactogenicity, and Immunogenicity of mRNA-1283 and mRNA-1273 Vaccines in Healthy Adults Between 18 Years and 55 Years of Age to Prevent COVID-19	IM	mRNA-1283 vs mRNA-1273	18 Years to 55 Years	1	125	2021-03-11
NCT04806113	COVID-19 Vaccine in Immunosuppressed Adults With Autoimmune Diseases	IM	mRNA-1273	18 Years and older	3	220	2021-03-11
NCT04798027	Study of mRNA Vaccine Formulation Against COVID-19 in Healthy Adults 18 Years of Age and Older	IM	RNA vaccine	18 Years and older	1 & 2	333	2021-03-12
NCT04821674	Study of DS-5670a (COVID-19 Vaccine) in Japanese Healthy Adults and Elderly Subjects	IM	DS-5670a	20 Years to 74 Years	1 & 2	152	2021-03-15
NCT04796896	A Study to Evaluate Safety and Effectiveness of mRNA-1273 Vaccine in Healthy Children Between 6 Months of Age and Less Than 12 Years of Age	IM	mRNA-1273	6 Months to 11 Years	2 & 3	7050	2021-03-15
NCT04816643	Study to Evaluate the Safety, Tolerability, and Immunogenicity of an RNA Vaccine Candidate Against COVID-19 in Healthy Children <12 Years of Age	UNS	BNT162b2	6 Months to 11 Years	1 & 2	4644	2021-03-24
NCT04785144	Safety and Immunogenicity Study of a SARS-CoV-2 (COVID-19) Variant Vaccine (mRNA-1273.351) in Na??ve and Previously Vaccinated Adults	IM	mRNA-1273 vs mRNA-1273.351	18 Years to 99 Years	1	210	2021-03-29
NCT04816669	Study to Evaluate the Safety, Tolerability, and Immunogenicity of Multiple Formulations of BNT162b2 Against COVID-19 in Healthy Adults	IM	BNT162b2	18 Years to 55 Years	3	610	2021-04-01
NCT04818736	COVID-19 Vaccine For Indirect Protection	UNS	mRNA-1273	Child, Adult, Older Adult	4	0	2021-04-15
NCT04860297	A Study to Evaluate Safety and Immunogenicity of mRNA-1273 Vaccine to Prevent COVID-19 in Adult Organ Transplant Recipients and in Healthy Adult Participants	IM	mRNA-1273	18 Years and older	3	240	2021-04-16
NCT04852978	COVID-19 Study to Assess Immunogenicity, Safety, and Tolerability of Moderna mRNA-1273 Vaccine Administered With Casirivimab+Imdevimab in Healthy Adult Volunteers	IM	mRNA-1273	18 Years to 90 Years	2	180	2021-04-20
NCT04860258	A Study to Evaluate Safety, Reactogenicity and Immunogenicity of the SARS-CoV-2 mRNA Vaccine CVnCoV in Adults With Co-morbidities for COVID-19	IM	CVnCoV	18 Years and older	3	1200	2021-04-22
NCT04860739	Vaccination With COMIRNATY in Subjects With a VAXZEVRIA First Dose	UNS	BNT162b2	18 Years to 60 Years	2	676	2021-04-24
NCT04863131	Safety and Immunogenicity of EXG-5003	ID	EXG-5003	20 Years to 55 Years	1 & 2	60	2021-04-28

		NCT04847050	A Trial of the Safety and Immunogenicity of the COVID-19 Vaccine (mRNA-1273) in Participants With Hematologic Malignancies and Various Regimens of Immunosuppression, and in Participants With Solid Tumors on PD1/PDL1 Inhibitor Therapy	IM	mRNA-1273	18 Years and older	2	120	2021-04-28
		NCT04878796	Effectiveness of mRNA Covid-19 Vaccines on Cancer Patients:Observational Study. (ANTICOV)	UNS	mRNA-1273 vs BNT162b2	18 Years and older	N/A	300	2021-05-01
		NCT04844268	Phase 1 Study to Assess Safety, Reactogenicity and Immunogenicity of the HDT-301 Vaccine Against COVID-19	IM	HDT 301	18 Years to 55 Years	1	78	2021-05-01
		NCT04848467	COVID-19: A Trial Studying the SARS-CoV-2 mRNA Vaccine CVnCoV to Learn About the Immune Response, the Safety, and the Degree of Typical Vaccination Reactions When CVnCoV is Given at the Same Time as a Flu Vaccine Compared to When the Vaccines Are Separately Given in Adults 60 Years of Age and Older	IM	CVnCoV	60 Years and older	3	1000	2021-05-10
		NCT04852861	COVID-19: Safety and Immunogenicity of a Reduced Dose of the BioNTech/Pfizer BNT162b2 Vaccine	UNS	BNT162b2	18 Years to 55 Years	4	150	2021-05-10
		NCT04838847	A Study to Evaluate the Immunogenicity and Safety of the SARS-CoV-2 mRNA Vaccine CVnCoV in Elderly Adults Compared to Younger Adults for COVID-19	IM	CVnCoV	18 Years and older	3	180	2021-05-14
		NCT04848584	Pfizer-BioNTech COVID-19 BNT162b2 Vaccine Effectiveness Study - Kaiser Permanente Southern California	UNS	BNT162b2	16 Years and older	N/A	999	2021-05-15
		NCT04889209	Efficacy Study of Phase 1/2 Randomized Interventional Study of SARS-COV-2-Vaccine Candidates Utilizing EUA Dosing	IM	mRNA-1273	18 Years to 99 Years	1 & 2	400	2021-05-17
		NCT04885907	Third Dose of Moderna in Transplant Recipients	UNS	mRNA-1273	18 Years and older	4	120	2021-05-17
Active immunity		NCT04869358	Exploring the Immune Response to SARS-CoV-2 COVID-19 Vaccines in Patients With Relapsing Multiple Sclerosis (RMS) Treated With Ofatumumab	UNS	RNA vaccine	18 Years to 100 Years	4	40	2021-05-24
		NCT04847102	A Phase III Clinical Study of a SARS-CoV-2 Messenger Ribonucleic Acid (mRNA) Vaccine Candidate Against COVID-19 in Population Aged 18 Years and Above	IM	RNA vaccine	18 Years and older	3	28000	2021-05-28
		NCT04892888	Survey of the Moderna COVID-19 Vaccine in People at High-Risk of Developing Severe COVID-19 Symptoms	IM	mRNA-1273	Child, Adult, Older Adult	N/A	1000	2021-06-01
		NCT04336410	Safety, Tolerability and Immunogenicity of INO-4800 for COVID-19 in Healthy Volunteers	ID	INO-4800	18 Years to 50 Years	1	40	2020-04-03
		NCT04445389	Safety and Immunogenicity Study of GX-19, a COVID-19 Preventive DNA Vaccine in Healthy Adults	IM	GX-19	18 Years to 50 Years	1 & 2	210	2020-06-17
		NCT04463472	Study of COVID-19 DNA Vaccine (AG0301-COVID19)	IM	AG0301-COVID19	20 Years to 65 Years	1 & 2	30	2020-06-29
		NCT04447781	Safety, Tolerability and Immunogenicity of INO-4800 Followed by Electroporation in Healthy Volunteers for COVID19	ID	INO-4800	19 Years to 64 Years	1 & 2	160	2020-07-15
		NCT04527081	Study of COVID-19 DNA Vaccine (AG0302-COVID19)	IM	AG0302-COVID19	20 Years to 65 Years	1 & 2	30	2020-08-31
		NCT04642638	Safety, Immunogenicity, and Efficacy of INO-4800 for COVID-19 in Healthy Seronegative Adults at High Risk of SARS-CoV-2 Exposure	ID	INO-4800	18 Years and older	2 & 3	6578	2020-11-01
	DNA vaccine	NCT04655625	Phase II / III Study of COVID-19 DNA Vaccine (AG0302-COVID19)	IM	AG0302-COVID19	18 Years and older	2 & 3	500	2020-11-23
		NCT04627675	CORVax12: SARS-CoV-2 Spike (S) Protein Plasmid DNA Vaccine Trial for COVID-19 (SARS-CoV-2)	Electroporation	CORVax	18 Years and older	1	36	2020-12-01
		NCT04591184	A Clinical Trial of a Plasmid DNA Vaccine for COVID-19 [Covigenix VAX-001] in Adults	IM	BVAX-001	18 Years to 84 Years	1	72	2020-12-01

NCT04673149	GLS-5310 Vaccine for the Prevention of SARS-CoV-2 (COVID-19)	IC	GLS-5310	19 Years to 65 Years	1 & 2	345	2020-12-23
NCT04715997	Safety and Immunogenicity Study of GX-19N, a COVID-19 Preventive DNA Vaccine in Healthy Adults	IM	GX-19N	18 Years to 55 Years	1 & 2	170	2020-12-30
NCT04742842	The Safety and Immunogenicity of a DNA-based Vaccine (COVIGEN) in Healthy Volunteers	IM	COVIGEN	18 Years to 75 Years	1	150	2021-02-15
NCT04788459	Safety and Immunogenicity of COVID-eVax, a Candidate Plasmid DNA Vaccine for COVID-19, in Healthy Adult Volunteers	IM	COVID-eVax	18 Years to 65 Years	1 & 2	160	2021-02-25
NCT04864561	Study To Compare The Immunogenicity Against COVID-19, Of VLA2001 Vaccine To AZD1222 Vaccine	IM	AZD1222 vs VLA2001	18 Years and older	3	4000	2021-04-26
NCT04892459	Study on Sequential Immunization of Inactive SARS-CoV-2 Vaccine and Recombinant SARS-CoV-2 Vaccine (Ad5 Vector)	UNS	SARS-CoV-2 Ad5 vs Inactivated SARS-CoV-2	18 Years to 59 Years	4	300	2021-05-19
NCT04313127	Phase I Clinical Trial of a COVID-19 Vaccine in 18-60 Healthy Adults	IM	Ad5-nCoV	18 Years to 60 Years	1	108	2020-03-16
NCT04341389	A Phase II Clinical Trial to Evaluate the Recombinant Vaccine for COVID-19 (Adenovirus Vector)	IM	Ad5-nCoV	18 Years and older	2	500	2020-04-12
NCT04324606	A Study of a Candidate COVID-19 Vaccine (COV001)	IM	AZD1222	18 Years to 55 Years	1 & 2	1112	2020-04-20
NCT04398147	Phase I/II Clinical Trial of Recombinant Novel Coronavirus Vaccine (Adenovirus Type 5 Vector) in Canada	IM	Ad5-nCoV	18 Years to 84 Years	1 & 2	696	2020-05-01
NCT04400838	Investigating a Vaccine Against COVID-19	IM	AZD1222	5 Years and older	2 & 3	12330	2020-05-28
NCT04536051	A Study of a Candidate COVID-19 Vaccine (COV003)	IM	AZD1222	18 Years and older	3	10300	2020-06-02
NCT04436471	An Open Study of the Safety, Tolerability and Immunogenicity of the Drug "Gam-COVID-Vac" Vaccine Against COVID-19	IM	Gam-COVID-Vac	18 Years to 60 Years	1 & 2	38	2020-06-17
NCT04437875	An Open Study of the Safety, Tolerability and Immunogenicity of "Gam-COVID-Vac Lyo" Vaccine Against COVID-19	IM	Gam-COVID-Vac Lyo	18 Years to 60 Years	1 & 2	38	2020-06-17
NCT04444674	COVID-19 Vaccine (ChAdOx1 nCoV-19) Trial in South African Adults With and Without HIV-infection	IM	AZD1222	18 Years to 65 Years	1 & 2	2000	2020-06-24
NCT04436276	A Study of Ad26.COV2.S in Adults (COVID-19)	IM	Ad26.COV2.S	18 Years and older	1 & 2	1045	2020-07-15
NCT04528641	GRAd-COV2 Vaccine Against COVID-19	IM	GRAd-COV2	18 Years to 85 Years	1	90	2020-08-10
NCT04497298	Clinical Trial to Evaluate the Safety and Immunogenicitiy of the COVID-19 Vaccine	IM	TMV-083	18 Years to 55 Years	1	90	2020-08-10
NCT04509947	A Study of Ad26.COV2.S in Adults (COVID-19)	IM	Ad26.COV2.S	20 Years and older	1	250	2020-08-11
NCT04516746	Phase III Double-blind, Placebo-controlled Study of AZD1222 for the Prevention of COVID-19 in Adults	IM	AZD1222	18 Years and older	3	30000	2020-08-17
NCT04568031	Study of AZD1222 for the Prevention of COVID-19 in Japan	IM	AZD1222	18 Years and older	1 & 2	256	2020-08-23
NCT04526990	Phase III Trial of A COVID-19 Vaccine of Adenovirus Vector in Adults 18 Years Old and Above	IM	Ad5-nCoV	18 Years and older	3	40000	2020-08-26
NCT04498247	A Study to Assess Safety, Tolerability, and Immunogenicity of V591 (COVID-19 Vaccine) in Healthy Participants (V591-001)	IM	V591	18 Years and older	1 & 2	260	2020-08-27
NCT04530396	Clinical Trial of Efficacy, Safety, and Immunogenicity of Gam-COVID-Vac Vaccine Against COVID-19	IM	Gam-COVID-Vac	18 Years to 111 Years	3	40000	2020-08-31
NCT04540393	AZD1222 Vaccine for the Prevention of COVID-19	IM	AZD1222	18 Years to 130 Years	3	100	2020-09-02

Viral vector vaccine	NCT04505722	A Study of Ad26.COV2.S for the Prevention of SARS-CoV-2-Mediated COVID-19 in Adult Participants	IM	Ad26.COV2.S	18 Years and older	3	60000	2020-09-05
	NCT04540419	Clinical Trial of Recombinant Novel Coronavirus Vaccine (Adenovirus Type 5 Vector) Against COVID-19	IM	Ad5-nCoV	18 Years to 85 Years	3	500	2020-09-11
	NCT04563702	Safety and Immunogenicity Trial of an Oral SARS-CoV-2 Vaccine (VXA-CoV2-1) for Prevention of COVID-19 in Healthy Adults	ORAL	VXA-CoV2-1	18 Years to 54 Years	1	35	2020-09-21
	NCT04566770	A Clinical Trial of A COVID-19 Vaccine Named Recombinant Novel Coronavirus Vaccine (Adenovirus Type 5 Vector)	IM	Ad5-nCoV	6 Years and older	2	481	2020-09-24
	NCT04568811	The Phase I Clinical Trial of Booster Vaccination of Adenovirus Type-5 Vectored COVID-19 Vaccine	UNS	Ad5-nCoV	Child, Adult, Older Adult	1	89	2020-09-26
	NCT04564716	Clinical Trial of Efficacy, Safety, and Immunogenicity of Gam-COVID-Vac Vaccine Against COVID-19 in Belarus	IM	Gam-COVID-Vac	18 Years to 60 Years	3	100	2020-09-28
	NCT04552366	A Clinical Trial of a Recombinant Adenovirus 5 Vectored COVID-19 Vaccine (Ad5-nCoV) With Two Doses in Healthy Adults	IM	Ad5-nCoV	18 Years and older	1	144	2020-09-29
	NCT04569383	Safety, Tolerability and Immunogenicity of the Candidate Vaccine MVA-SARS-2-S Against COVID-19	UNS	MVA-SARS-2-S	18 Years to 55 Years	1	30	2020-10-01
	NCT04591717	Study of the Safety of Prophylactic Vaccination With 2nd Generation E1/E2B/E3-Deleted Adenoviral-COVID-19 in Normal Healthy Volunteers	UNS	hAd5-S-Fusion+N-ETSD	18 Years to 55 Years	1	35	2020-10-19
	NCT04587219	The Study of "Gam-COVID-Vac" Vaccine Against COVID-19 With the Participation of Volunteers of 60 y.o and Older	IM	Gam-COVID-Vac	60 Years to 111 Years	2	110	2020-10-22
	NCT04608305	Evaluate the Safety, Immunogenicity and Potential Efficacy of an rVSV-SARS-CoV-2-S Vaccine	UNS	IIBR-100	18 Years to 85 Years	1 & 2	1040	2020-10-28
	NCT04569786	Dose Ranging Trial to Assess Safety and Immunogenicity of V590 (COVID-19 Vaccine) in Healthy Adults (V590-001)	IM	V590	18 Years and older	1	252	2020-10-30
	NCT04642339	Clinical Trial of the Immunogenicity, Safety, and Efficacy of the Gam-COVID-Vac Vaccine Against COVID-19 in Venezuela	IM	Gam-COVID-Vac	18 Years and older	3	2000	2020-11-01
	NCT04614948	A Study of Ad26.COV2.S for the Prevention of SARS-CoV-2-mediated COVID-19 in Adults	IM	Ad26.COV2.S	18 Years and older	3	30000	2020-11-15
	NCT04639466	A Synthetic MVA-based SARS-CoV-2 Vaccine, COH04S1, for the Prevention of COVID-19	IM	COH04S1	18 Years to 55 Years	1	129	2020-11-25
	NCT04640233	Clinical Trial to Assess Safety and Immunogenicity of Gam-COVID-Vac Combined Vector Vaccine for Severe Acute Respiratory Syndrome Coronavirus 2 (SARS-茶ov-2) Infection	IM	Gam-COVID-Vac	18 Years and older	2 & 3	1600	2020-12-01
	NCT04656613	A Phase III Clinical Trial of the Immunogenicity and Safety of the Gam-COVID-Vac Vaccine Against COVID-19 in the UAE	IM	Gam-COVID-Vac	18 Years and older	3	1000	2020-12-01
	NCT04666012	Safety and Immunogenicity Study of AdCLD-CoV19: A COVID-19 Preventive Vaccine in Healthy Volunteers	IM	AdCLD-CoV19	19 Years to 64 Years	1 & 2	150	2020-12-29
	NCT04738435	Safety of the Sputnik V Vaccine in Health Personnel of Private Effectors of the City of Buenos Aires, Argentina	UNS	Sputnik V	18 Years and older	N/A	600	2021-01-05
	NCT04723602	Evaluation of Safety, Tolerability and Immune Responses of Ebola-S and Marburg Vaccines in Healthy Adults	IM	cAd3-EBO-S	18 Years to 50 Years	1	32	2021-01-06
	NCT04713488	An Open Study on the Safety, Tolerability, and Immunogenicity of "Sputnik Light" Vaccine	IM	Sputnik Light	18 Years to 111 Years	1 & 2	110	2021-01-15
	NCT04679909	Safety and Immunogenicity of AdCOVID in Healthy Adults (COVID-19 Vaccine Study)	IN	Biological: AdCOVID|Other: Placebo	18 Years to 55 Years	1	180	2021-02-01

	NCT04730895	Isotretinoin (13- Cis-Retinoic Acid) Versus Spike Protein Based Vaccine for Providing Complete Protection Against COVID-19 and Preventing the Expected Long Term Serious Side Effects Which May Caused by Spike Protein Based Vaccine-An Urgent Protective Clinical Trial	UNS	AZD1222	18 Years to 40 Years	1 & 2	360	2021-02-01
	NCT04741061	Study to Evaluate Efficacy, Immunogenicity and Safety of the Sputnik-Light	UNS	Sputnik Light	18 Years to 111 Years	3	6000	2021-02-19
	NCT04732468	COVID-19 Oral and Subcutaneous Vaccination Using a 2nd Generation (E1/E2B/E3-Deleted) Adenovirus Platform in Healthy Volunteers in USA	SC	hAd5-S-Fusion+N-ETSD	18 Years to 55 Years	1	65	2021-02-24
	NCT04751682	Safety and Immunogenicity of an Intranasal SARS-CoV-2 Vaccine (BBV154) for COVID-19	IN	BBV154	18 Years to 60 Years	1	175	2021-03-01
	NCT04809948	Effects of COVID 19 Vaccine on Egyptian Population	UNS	Viral vector	18 Years to 80 Years	N/A	800	2021-03-01
	NCT04710303	COVID-19 Vaccination Using a 2nd Generation (E1/E2B/E3-Deleted) Adenoviral Platform in Healthy South African Adults	UNS	hAd5-S-Fusion+N-ETSD	18 Years to 50 Years	1	35	2021-03-02
	NCT04830800	A Phase 1/2 Safety and Immunogenicity Trial of COVID-19 Vaccine COVIVAC	IM	COVIVAC	18 Years to 75 Years	1 & 2	420	2021-03-10
	NCT04765384	A Study of Ad26.COV2.S in Healthy Pregnant Participants (COVID-19)	IM	Ad26.COV2.S	18 Years to 45 Years	2	400	2021-03-12
	NCT04760730	Safety and Immunogenicity Study in Adults of AZD1222 and rAd26-S Administered as Heterologous Prime Boost Regimen for the Prevention of Coronavirus Disease 2019 (COVID-19)	IM	AZD1222	18 Years to 100 Years	1 & 2	100	2021-03-17
	NCT04794946	Safety and Efficacy of a Non-replicating ChAdOx1 Vector Vaccine AZD1222 (COVISHIELD), for Prevention of COVID-19 in Patients With Liver Cirrhosis	UNS	AZD1222	18 Years to 60 Years	N/A	2200	2021-03-19
	NCT04764422	Assess the Safety and Immunogenicity of NDV-HXP-S Vaccine in Thailand	IM	NDV-HXP-S	18 Years to 75 Years	1 & 2	460	2021-03-20
	NCT04809389	A Study to Evaluate Safety and Immunogenicity of DelNS1-nCoV-RBD LAIV for COVID-19	IN	DelNS1-nCoV-RBD LAIV	18 Years to 55 Years	1	115	2021-03-29
	NCT04684446	AstraZeneca Vax - Sputnik V Combination Study	IM	AZD1222	18 Years to 130 Years	1 & 2	100	2021-03-30
	NCT04816019	A Study of Intranasal ChAdOx1 nCOV-19	IN	AZD1222	18 Years to 40 Years	1	54	2021-04-01
	NCT04871841	Study of Sputnik V COVID-19 Vaccination in Adults in Kazakhstan	IM	Sputnik V	18 Years and older	N/A	70	2021-04-05
	NCT04798001	Safety and Immunogenicity of an Intranasal RSV Vaccine Expressing SARS-CoV-2 Spike Protein (COVID-19 Vaccine) in Adults	IN	MV-014-212	18 Years to 69 Years	1	130	2021-04-12
	NCT04840992	Phase I/II Clinical Trial of Recombinant Novel Coronavirus (COVID-19) Vaccine (Adenovirus Type 5 Vector) for Inhalation	IM	Ad5-nCoV	18 Years and older	1 & 2	840	2021-04-20
	NCT04871737	Study of a Live rNDV Based Vaccine Against COVID-19	IM	Newcastle Disease virus vector	18 Years to 55 Years	1	90	2021-05-01
	NCT04894305	A Study of Ad26.COV2.S in Healthy Adults (COVID-19)	IM	Ad26.COV2.S	18 Years to 65 Years	1	380	2021-05-14
	NCT04839042	A Phase 1, First-In-Human Study of the Investigational COVID-19 Vaccine SC-Ad6-1 in Healthy Volunteers	IM	SC-Ad6-1	18 Years to 55 Years	1	40	2021-06-01
Bacterial vector vaccine	NCT04334980	Evaluating the Safety, Tolerability and Immunogenicity of bacTRL-Spike Vaccine for Prevention of COVID-19	ORAL	bacTRL-Spike	19 Years to 45 Years	1	84	2020-04-30
	NCT04352608	Safety and Immunogenicity Study of 2019-nCoV Vaccine (Inactivated) for Prophylaxis SARS CoV-2 Infection (COVID-19)	UNS	Inactivated	18 Years to 59 Years	1 & 2	744	2020-04-13
	NCT04412538	Safety and Immunogenicity Study of an Inactivated SARS-CoV-2 Vaccine for Preventing Against COVID-19	UNS	Inactivated	18 Years to 59 Years	1 & 2	942	2020-05-15

	NCT04383574	Safety and Immunogenicity Study of Inactivated Vaccine for Prevention of SARS-CoV-2 Infection(COVID-19)	UNS	Inactivated	60 Years and older	1 & 2	422	2020-05-20
	NCT04380532	Tableted COVID-19 Therapeutic Vaccine	ORAL	V-SARS	18 Years to 65 Years	1 & 2	20	2020-06-15
	NCT04470609	Safety and Immunogenicity Study of an Inactivated SARS-CoV-2 Vaccine for Preventing Against COVID-19 in People Aged 激?0 Years	UNS	Inactivated	60 Years and older	1 & 2	471	2020-07-10
	NCT04471519	Whole-Virion Inactivated SARS-CoV-2 Vaccine (BBV152) for COVID-19 in Healthy Volunteers	IM	BBV152	12 Years to 65 Years	1 & 2	1125	2020-07-13
	NCT04510207	A Study to Evaluate The Efficacy, Safety and Immunogenicity of Inactivated SARS-CoV-2 Vaccines (Vero Cell) in Healthy Population Aged 18 Years Old and Above	UNS	Inactivated (Vero cell)	18 Years and older	3	45000	2020-07-16
	NCT04456595	Clinical Trial of Efficacy and Safety of Sinovac's Adsorbed COVID-19 (Inactivated) Vaccine in Healthcare Professionals	IM	Inactivated	18 Years and older	3	8870	2020-07-21
	NCT04508075	Efficacy, Safety and Immunogenicity Study of SARS-CoV-2 Inactivated Vaccine	IM	Inactivated	18 Years to 59 Years	3	1620	2020-08-10
	NCT04530357	Reactogenicity, Safety and Immunogenicity of QazCovid-in® COVID-19 Vaccine	IM	QazCovid-in®	18 Years to 100 Years	1 & 2	244	2020-09-01
	NCT04612972	Efficacy, Safety and Immunogenicity of Inactivated SARS-CoV-2 Vaccines (Vero Cell) in Healthy Adult Population In Peru	UNS	Inactivated (Vero cell)	18 Years to 60 Years	3	6000	2020-09-10
	NCT04582344	Clinical Trial For SARS-CoV-2 Vaccine (COVID-19)	IM	CoronaVac	18 Years to 59 Years	3	13000	2020-09-14
	NCT04560881	Clinical Trial to Evaluate the Efficacy, Immunogenicity and Safety of the Inactivated SARS-CoV-2 Vaccine (COVID-19)	IM	Inactivated (Vero cell)	18 Years to 85 Years	3	3000	2020-09-16
	NCT04758273	A Safety and Immunogenicity Study of Inactivated SARS-CoV-2 Vaccine (Vero Cells) in Healthy Population Aged 18 Years and Above(COVID-19)	UNS	Inactivated	18 Years and older	1	180	2020-10-07
	NCT04756323	A Study to Evaluate Safety and Immunogenicity of Inactivated SARS-CoV-2 Vaccine (Vero Cells) in Healthy Population Aged 18 Years and Above(COVID-19)	UNS	Inactivated	18 Years and older	2	1000	2020-10-27
	NCT04617483	Study of the Commercial Scale SARS-CoV-2 Vaccine Against the Pilot Scale Among Adults, and Bridging Study of the Immunogenicity in Elderly Against That in Adults	UNS	Inactivated	18 Years and older	3	1040	2020-10-31
	NCT04551547	Safety and Immunogenicity Study of Inactivated Vaccine for Prevention of COVID-19	UNS	Inactivated	3 Years to 17 Years	1 & 2	552	2020-10-31
	NCT04691947	Safety and Immunogenicity of Two Different Strengths of the Inactivated COVID-19 Vaccine ERUCOV-VAC	IM	ERUCOV-VAC	18 Years to 55 Years	1	44	2020-11-05
Inactivated virus vaccine	NCT04641481	An Efficacy and Safety Clinical Trial of an Investigational COVID-19 Vaccine (BBV152) in Adult Volunteers	UNS	BBV152	18 Years to 99 Years	3	25800	2020-11-25
	NCT04651790	Efficacy, Safety, and Immunogenicity of Two Vaccination Schedules of an Inactivated Vaccine Against COVID-19 in Adults	UNS	Inactivated	18 Years and older	3	2300	2020-11-27
	NCT04691908	Immunogenicity, Efficacy and Safety of QazCovid-in?? COVID-19 Vaccine	IM	QazCovid-in®	18 Years and older	3	3000	2020-12-25
	NCT04795414	Safety and Immunogenicity of an Inactivated Vaccine Against COVID-19 in Medical Workers	UNS	Inactivated (Vero cell)	18 Years to 59 Years	N/A	1370	2021-01-14
	NCT04659239	The Efficacy, Safety and Immunogenicity Study of Inactivated SARS-CoV-2 Vaccine for Preventing Against COVID-19	UNS	Inactivated (Vero cell)	18 Years and older	3	34020	2021-01-28
	NCT04756830	A Study to Assess the Safety and Immunogenicity of the Coronavac Vaccine Against COVID-19	UNS	Inactivated	18 Years and older	4	1200	2021-02-01
	NCT04747821	An Effectiveness Study of the Sinovac's Adsorbed COVID-19 (Inactivated) Vaccine	UNS	Inactivated	18 Years and older	4	27711	2021-02-07
	NCT04754698	COVID-19 CoronaVac in Patients With Autoimmune Rheumatic Diseases and HIV/AIDS	UNS	CoronaVac	18 Years and older	4	2067	2021-02-09

	NCT04824391	Efficacy, Immunogenicity and Safety of Inactivated ERUCOV-VAC Compared With Placebo in COVID-19	IM	ERUCOV-VAC	18 Years to 64 Years	2	250	2021-02-10
	NCT04756271	Safety and Immunogenicity of Inactivated Vaccine Against COVID-19 Infection	UNS	Inactivated	18 Years and older	3	140	2021-02-11
	NCT04790851	Evaluation of Immunogenicity and Safety of Combined Immunization of COVIV and PPV23 / IIV4	UNS	COVIV	18 Years and older	4	1152	2021-02-23
	NCT04801888	Study on Combined Vaccination With SARS-CoV-2 Inactivated Vaccine and Quadrivalent Influenza Vaccine	UNS	Inactivated	18 Years to 59 Years	4	480	2021-03-01
	NCT04854408	Evaluation of the Effect of Coronavac Vaccine (Severe Acute Respiratory Syndrome Coronavirus 2 (SARS-CoV-2 Vaccine) on Healthcare Workers' Menstrual Patterns	UNS	CoronoVAC	18 Years to 45 Years	N/A	300	2021-03-18
	NCT04789356	Effectiveness of the Adsorbed Vaccine COVID-19 (Coronavac) Among Education and Public Safety Workers With Risk Factors for Severity	IM	Inactivated	18 Years to 49 Years	4	10156	2021-03-18
	NCT04838080	Safety and Immunogenicity of the Inactivated Koç ak-19 Inaktif Adjuvanlı COVID-19 Vaccine Compared to Placebo	IM	Koçak-19 Inaktif Adjuvanl	18 Years to 55 Years	1	38	2021-03-19
	NCT04765215	Investigation of the Effectiveness of CoronaVac Vaccine in Cancer Patients With Active Chemotherapy and Comparison With Healthy People.	UNS	CoronoVAC	18 Years to 90 Years	N/A	291	2021-03-31
	NCT04866069	Study of a Severe Acute Respiratory Syndrome-Coronavirus-2 (SARS-CoV-2) Adjuvanted Inactivated Vaccine in Healthy Adults	SC	Inactivated	18 Years to 45 Years	1	50	2021-04-25
	NCT04863638	A Immuno-bridging and Immunization Schedules Study of COVID-19 Vaccine (Vero Cell), Inactivated	UNS	Inactivatet (vero cell)	3 Years and older	4	4400	2021-04-29
	NCT04852705	A Study to Evaluate the Efficacy, Safety and Immunogenicity of SARS-CoV-2 Vaccine (Vero Cells), Inactivated in Healthy Adults Aged 18 Years and Older (COVID-19)	IM	Inactivatet (vero cell)	18 Years and older	3	28000	2021-05-01
	NCT04884685	Safety of an Inactivated SARS-CoV-2 Vaccine (CoronaVac) in Children and Adolescents	IM	Inactivated	3 Years to 17 Years	2	500	2021-05-03
	NCT04450004	Safety, Tolerability and Immunogenicinity of a Coronavirus-Like Particle COVID-19 Vaccine in Adults Aged 18-55 Years.	IM	Coronavirus-Like Particle	18 Years to 55 Years	1	180	2020-07-10
	NCT04636697	Study of a Recombinant Coronavirus-Like Particle COVID-19 Vaccine in Adults	IM	Coronavirus-Like Particle	18 Years and older	2 & 3	30612	2020-11-19
	NCT04839146	Safety and Tolerability of COVID-19 Vaccine (ABNCoV2)	IM	ABNCoV2 Vaccine	18 Years to 55 Years	1	42	2021-03-11
	NCT04773665	Safety, Tolerability, and Immunogenicity of the COVID-19 Vaccine Candidate (VBI-2902a)	IM	VBI-2902a	18 Years and older	1 & 2	780	2021-03-15
	NCT04818281	Study of a Severe Acute Respiratory Syndrome CoV-2 (SARS-CoV-2) Virus-like Particle (VLP) Vaccine in Healthy Adults	SC	SARS-CoV-2 VLP	18 Years to 59 Years	1	36	2021-03-27
Virus-like particle	NCT04619628	Safety and Immunogenicity of COVI-VAC, a Live Attenuated Vaccine Against COVID-19	IV	COVI-VAC	18 Years to 30 Years	1	48	2020-12-01
	NCT04261426	The Efficacy of Intravenous Immunoglobulin Therapy for Severe 2019-nCoV Infected Pneumonia	IV	IVIG	18 Years and older	2 & 3	80	2020-02-10
	NCT04350580	Polyvalent Immunoglobulin in COVID-19 Related ARds	IV	IVIG	18 Years and older	3	138	2020-04-11
	NCT04411667	Study of Standard of Care Plus Intravenous Immunoglobulin (IVIG) Compared to Standard of Care Alone in the Treatment of COVID-19 Infection	IV	Octagam	18 Years and older	4	40	2020-04-28
	NCT04500067	Intravenous Immunoglobulin (IVIG, Bioven) Efficacy Assess for COVID-19 / SARS-CoV-2 Severe Pneumonia Complex Treatment	IV	IVIG	18 Years and older	3	76	2020-05-07
	NCT04432324	Study to Evaluate the Safety and Efficacy of High Dose IVIG in Hospitalized Participants With Coronavirus Disease (COVID-19)	IV	IVIG	18 Years and older	2	100	2020-06-01

	NCT04400058	Octagam 10% Therapy in COVID-19 Patients With Severe Disease Progression	IV	Octagam	18 Years and older	3	208	2020-06-01
	NCT04429529	Safety of TY027, a Treatment for COVID-19, in Humans	IV	TY027	21 Years to 50 Years	1	25	2020-06-09
	NCT04521309	SARS-CoV-2 Antibodies Based IVIG Therapy for COVID-19 Patients	IV	IVIG	18 Years and older	1 & 2	50	2020-06-19
Immunoglobulin	NCT04480424	Study to Evaluate the Safety and Efficacy of High Dose Intravenous Immune Globulin (IVIG) Plus Standard Medical Treatment (SMT) Versus SMT Alone in Participants in Intensive Care Unit (ICU) With Coronavirus Disease (COVID-19)	IV	GAMUNEX-C	18 Years and older	2	100	2020-07-01
	NCT04469179	Safety, Tolerability, and Pharmacokinetics of SAB-185 in Ambulatory Participants With COVID-19	IV	SAB-185	18 Years to 60 Years	1	21	2020-07-01
	NCT04468958	Safety, Tolerability, and Pharmacokinetics of SAB-185 in Healthy Participants	IV	SAB-185	18 Years to 60 Years	1	28	2020-08-05
	NCT04610502	Efficacy and Safety of Two Hyperimmune Equine Anti Sars-CoV-2 Serum in COVID-19 Patients	IV	Equine immunoglobulin	18 Years and older	2	26	2020-09-06
	NCT04548557	Intravenous Immunoglobulins for the Treatment of Covid-19 Patients: a Clinical Trial	IV	IVIG	18 Years to 90 Years	3	60	2020-09-15
	NCT04514302	Safety and Efficacy of Anti-SARS-CoV-2 Equine Antibody Fragments (INOSARS) for Hospitalized Patients With COVID-19	IV	Anti-SARS-CoV-2 equine immunoglobulin fragments (INOSARS)	18 Years and older	1 & 2	51	2020-10-20
	NCT04631666	COVID-19 - Administration of the SARS-CoV-2-Neutralizing Monoclonal Antibody DZIF-10c (Infusion)	IV	DZIF-10c	18 Years to 70 Years	1 & 2	69	2020-11-23
	NCT04631705	COVID-19 - Administration of the SARS-CoV-2-Neutralizing Monoclonal Antibody DZIF-10c (Inhalation)	ORAL	DZIF-10c	18 Years to 70 Years	1 & 2	69	2020-11-23
	NCT04649515	Efficacy and Safety of TY027, a Treatment for COVID-19, in Humans	IV	TY027	21 Years to 100 Years	3	1305	2020-12-04
	NCT04891172	Anti COVID 19 Intravenous Immunoglobulin (C-IVIG) Therapy for Severe COVID-19 Patients	IV	IVIG	18 Years and older	2 & 3	310	2021-06-01
	NCT04292340	Anti-SARS-CoV-2 Inactivated Convalescent Plasma in the Treatment of COVID-19	IV		Child, Adult, Older Adult	Safety & efficacy	15	2020-02-01
	NCT04321421	Hyperimmune Plasma for Critical Patients With COVID-19	IV		18 Years and older	N/A	49	2020-03-17
	NCT04389944	Amotosalen-Ultraviolet A Pathogen-Inactivated Convalescent Plasma in Addition to Best Supportive Care and Antiviral Therapy on Clinical Deterioration in Adults Presenting With Moderate to Severe COVID-19	IV		18 Years and older	N/A	15	2020-03-31
	NCT04332380	Convalescent Plasma for Patients With COVID-19: A Pilot Study	IV		18 Years to 60 Years	2	10	2020-04-01
	NCT04332835	Convalescent Plasma for Patients With COVID-19: A Randomized, Open Label, Parallel, Controlled Clinical Study	IV		18 Years to 60 Years	2 & 3	80	2020-04-01
	NCT04342182	Convalescent Plasma as Therapy for Covid-19 Severe SARS-CoV-2 Disease (CONCOVID Study)	IV		18 Years and older	2 & 3	426	2020-04-08
	NCT04347681	Potential Efficacy of Convalescent Plasma to Treat Severe COVID-19 and Patients at High Risk of Developing Severe COVID-19	IV		18 Years to 85 Years	2	40	2020-04-12
	NCT04333355	Safety in Convalescent Plasma Transfusion to COVID-19	IV		18 Years and older	1	20	2020-04-15
Passive immunity	NCT04345991	Efficacy of Convalescent Plasma to Treat COVID-19 Patients, a Nested Trial in the CORIMUNO-19 Cohort	IV		18 Years and older	2	120	2020-04-15
	NCT04352751	Experimental Use of Convalescent Plasma for Passive Immunization in Current COVID-19 Pandemic in Pakistan in 2020	IV		18 Years to 55 Years	N/A	2000	2020-04-20
	NCT04346446	Efficacy of Convalescent Plasma Therapy in Severely Sick COVID-19 Patients	IV		18 Years to 65 Years	2	20	2020-04-21

	NCT04362176	Passive Immunity Trial of Nashville II	IV	18 Years and older	3	500	2020-04-24
	NCT04476888	Convalescent Plasma Treatment in COVID-19	IV	18 Years and older	N/A	100	2020-04-26
	NCT04358783	Convalescent Plasma Compared to the Best Available Therapy for the Treatment of SARS-CoV-2 Pneumonia	IV	18 Years and older	2	30	2020-04-27
	NCT04348656	CONvalescent Plasma for Hospitalized Adults With COVID-19 Respiratory Illness (CONCOR-1)	IV	16 Years and older	3	1200	2020-04-27
	NCT04393727	Transfusion of Convalescent Plasma for the Early Treatment of Patients With COVID-19	IV	18 Years and older	2	126	2020-05-01
	NCT04377568	Efficacy of Human Coronavirus-immune Convalescent Plasma for the Treatment of COVID-19 Disease in Hospitalized Children	IV	up to 18 Years	2	100	2020-05-01
	NCT04407208	Convalescent Plasma Therapy in Patients With COVID-19	IV	18 Years and olde	1	10	2020-05-01
	NCT04565197	Convalescent Plasma Therapy for COVID-19 Patients	IV	15 Years to 80 Years	1	20	2020-05-01
Convalescent plasma	NCT04374565	Convalescent Plasma for Treatment of COVID-19 Patients With Pneumonia	IV	18 Years and older	2	29	2020-05-05
	NCT04374526	Early transfusIon of Convalescent Plasma in Elderly COVID-19 Patients. to Prevent Disease Progression.	IV	65 Years and older	2 & 3	182	2020-05-15
	NCT04442958	Effectiveness of Convalescent Immune Plasma Therapy	IV	18 Years to 90 Years	N/A	60	2020-05-15
	NCT04622826	plasmApuane CoV-2 : Efficacy and Safety of Immune Covid-19 Plasma in Covid-19 Pneumonia in Non ITU Patients	IV	18 Years and older	2	50	2020-05-15
	NCT04622826	plasmApuane CoV-2 : Efficacy and Safety of Immune Covid-19 Plasma in Covid-19 Pneumonia in Non ITU Patients	IV	18 Years and older	2	50	2020-05-15
	NCT04385186	Inactivated Convalescent Plasma as a Therapeutic Alternative in Patients CoViD-19	IV	18 Years and older	2	60	2020-05-20
	NCT04403477	Convalescent Plasma Therapy in Severe COVID-19 Infection	IV	16 Years and older	2	20	2020-05-20
	NCT04418518	A Trial of CONvalescent Plasma for Hospitalized Adults With Acute COVID-19 Respiratory Illness	IV	18 Years to 70 Years	3	1200	2020-06-01
	NCT04412486	COVID-19 Convalescent Plasma (CCP) Transfusion	IV	18 Years and older	1	100	2020-06-01
	NCT04373460	Convalescent Plasma to Limit SARS-CoV-2 Associated Complications	IV	18 Years and older	2	600	2020-06-03
	NCT04425915	Efficacy of Convalescent Plasma Therapy in Patients With COVID-19	IV	18 Years and older	3	400	2020-06-09
	NCT04323800	Convalescent Plasma to Stem Coronavirus (CSSC-001)	IV	18 Years and older	2	500	2020-06-10
	NCT04385186	Inactivated Convalescent Plasma as a Therapeutic Alternative in Patients CoViD-19	IV	18 Years and older	2	60	2020-06-20
	NCT04542967	Study on the Safety and Efficacy of Convalescent Plasma in Patients With Severe COVID-19 Disease	IV	18 Years to 90 Years	2	150	2020-06-23
	NCT04614012	Hyperimmune Plasma for Patients With COVID-19	IV	18 Years and older	N/A	100	2020-07-02
	NCT04803370	Efficacy of Reinforcing Standard Therapy in COVID-19 Patients With Repeated Transfusion of Convalescent Plasma	IV	18 Years and older	N/A	100	2020-07-08
	NCT04547660	Convalescent Plasma for Severe COVID-19 Patients	IV	18 Years and older	3	160	2020-07-16
	NCT04545047	Observational Study of Convalescent Plasma for Treatment of Veterans With COVID-19	IV	18 Years and older	N/A	4000	2020-08-07
	NCT04355767	Convalescent Plasma in Outpatients With COVID-19	IV	18 Years and older	3	600	2020-08-11
	NCT04621123	Plasma for Early Treatment in Non-hospitalised Mild or Moderate COVID-19 Patients	IV	50 Years and older	2	474	2020-10-30
	NCT04644198	Convalescent Plasma Transfusion in Severe COVID-19 Patients in Jamaica	IV	18 Years to 65 Years	2	30	2020-12-01

	NCT04712344	Assessment of Efficacy and Safety of Therapy With COVID-19 Convalescent Plasma in Subjects With Severe COVID-19 (IPCO)	IV		18 Years and older	2	58	2021-01-18
Neutralized antibody/inhibitor	NCT04148430	A Study of Anakinra to Prevent or Treat Severe Side Effects for Patients Receiving CAR-T Cell Therapy	SC	Anakinra	18 Years and older	2	90	2019-10-30
	NCT04288713	Eculizumab (Soliris) in Covid-19 Infected Patients	IV	Eculizumab	18 Years and older	N/A		2020-02-01
	NCT04306705	Tocilizumab vs CRRT in Management of Cytokine Release Syndrome (CRS) in COVID-19	IV	Tocilizumab	18 Years to 80 Years	N/A	120	2020-02-20
	NCT04324073	Cohort Multiple Randomized Controlled Trials Open-label of Immune Modulatory Drugs and Other Treatments in COVID-19 Patients - Sarilumab Trial - CORIMUNO-19 - SARI	IV	Sarilumab	18 Years and older	2 & 3	239	2020-03-27
	NCT04331808	CORIMUNO-19 - Tocilizumab Trial - TOCI (CORIMUNO-TOCI)	IV	Drug: Tocilizumab	18 Years and older	2	228	2020-03-30
	NCT04324021	Efficacy and Safety of Emapalumab and Anakinra in Reducing Hyperinflammation and Respiratory Distress in Patients With COVID-19 Infection.	IV	Biological: Emapalumab|Biological: Anakinra	30 Years to 79 Years	2 & 3	54	2020-04-02
	NCT04332094	Clinical Trial of Combined Use of Hydroxychloroquine, Azithromycin, and Tocilizumab for the Treatment of COVID-19	SC	Tocilizumab	18 Years and older	2	276	2020-04-02
	NCT04339712	Personalised Immunotherapy for SARS-CoV-2 (COVID-19) Associated With Organ Dysfunction	IV	Anakinra vs Tocilizumab	18 Years and older	2	102	2020-04-02
	NCT04322773	Anti-il6 Treatment of Serious COVID-19 Disease With Threatening Respiratory Failure	SC & IV	RoActemra	18 Years and older	2	200	2020-04-05
	NCT04361552	Tocilizumab for the Treatment of Cytokine Release Syndrome in Patients With COVID-19 (SARS-CoV-2 Infection)	IV	Tocilizumab	18 Years and older	3	0	2020-04-07
	NCT04341584	CORIMUNO-ANA: Trial Evaluating Efficacy Of Anakinra In Patients With Covid-19 Infection	IV	Anakinra	18 Years and older	2	161	2020-04-08
	NCT04341870	Study of Immune Modulatory Drugs and Other Treatments in COVID-19 Patients: Sarilumab, Azithromycin, Hydroxychloroquine Trial - CORIMUNO-19 - VIRO	IV	Sarilumab	18 Years to 80 Years	2 & 3	27	2020-04-11
	NCT04356508	COVID-19: A Pilot Study of Adaptive Immunity and Anti-PD1	IV	Nivolumab	18 Years and older	2	15	2020-04-14
	NCT04343144	Trial Evaluating Efficacy and Safety of Nivolumab (Optivo®) in Patients With COVID-19 Infection, Nested in the Corimmuno-19 Cohort.	IV	Nivolumab	18 Years and older	2	92	2020-04-15
	NCT04346797	CORIMUNO19-ECU: Trial Evaluating Efficacy and Safety of Eculizumab (Soliris) in Patients With COVID-19 Infection, Nested in the CORIMUNO-19 Cohort	IV	Eculizumab	18 Years to 75 Years	2	120	2020-04-16
	NCT04335071	Tocilizumab in the Treatment of Coronavirus Induced Disease (COVID-19)	SC	Tocilizumab	30 Years to 80 Years	2	100	2020-04-26
	NCT04371367	Avdoralimab an Anti-C5aR Antibody, in Patients With COVID-19 Severe Pneumonia (FORCE)	IV	avdoralimab	18 Years to 80 Years	2	108	2020-04-27
	NCT04351152	Phase 3 Study to Evaluate Efficacy and Safety of Lenzilumab in Hospitalized Patients With COVID-19 Pneumonia	IV	Lenzilumab	18 Years to 85 Years	3	238	2020-04-30
	NCT04560205	Tocilizumab in COVID-19 Lahore General Hospital	IV	Tocilizumab	15 Years to 80 Years	1	50	2020-05-01
	NCT04370834	Tocilizumab for Patients With Cancer and COVID-19 Disease	IV	Tocilizumab	2 Years and older	2	217	2020-05-28
	NCT04413838	Efficiency and Security of NIVOLUMAB Therapy in Obese Individuals With COVID-19(COrona VIrus Disease) Infection	IV	NIVOLUMAB	18 Years to 70 Years	2	120	2020-06-15
	NCT04494724	Clazakizumab vs. Placebo - COVID-19 Infection	IV	Clazakizumab	18 Years and older	2	60	2020-07-13
	NCT04540120	Safety and Efficacy of Dapansutrile for Treatment of Moderate COVID-19 Symptoms and Evidence of Early Cytokine Release Syndrome	ORAL	dapansutrile capsules	18 Years and older	2	80	2020-09-25
	NCT04583956	ACTIV-5 / Big Effect Trial (BET-A) for the Treatment of COVID-19	IV	Risankizumab	18 Years to 99 Years	2	200	2020-10-14

Immunotherapy		NCT04583969	ACTIV-5 / Big Effect Trial (BET-B) for the Treatment of COVID-19	IV	Lenzilumab	18 Years to 99 Years	2	200	2020-10-19
		NCT04600141	Clinical Efficacy of Heparin and Tocilizumab in Patients With Severe COVID-19 Infection: a Randomized Clinical Trial	IV	Tocilizumab	18 Years and older	3	308	2020-10-20
		NCT04734678	Comparison of Tocilizumab Versus Tocilizumab/Infliximab in Patients With COVID-19-associated Cytokine Storm Syndrome	IV	Tocilizumab vs Infliximab	18 Years to 65 Years	N/A	84	2020-12-01
		NCT04646603	MRG-001 as an Immunoregulatory and Regenerative Therapy for COVID-19 Patients	SC	MRG-001	18 Years to 45 Years	1 & 2	18	2021-01-28
		NCT04710199	Trial to Evaluate the Safety and Efficacy of Maraviroc in Patients Hospitalized for Coronavirus Disease 2019 (COVID-19)	ORAL	Maraviroc	18 Years and older	2	40	2021-02-08
	Cytokine	NCT04276688	Lopinavir/ Ritonavir, Ribavirin and IFN-beta Combination for nCoV Treatment	SC	Interferon Beta-1B	18 Years and older	2	127	2020-02-10
		NCT04385095	Trial of Inhaled Anti-viral (SNG001) for SARS-CoV-2 (COVID-19) Infection	Inhalation	Interferon beta 1a	18 Years and older	2	400	2020-03-16
		NCT04354259	Interferon Lambda for Immediate Antiviral Therapy at Diagnosis in COVID-19	UNS	Peginterferon Lambda-1A	18 Years and older	2	140	2020-05-13
		NCT04379076	InterLeukin-7 (CYT107) to Improve Clinical Outcomes in Lymphopenic pAtients With COVID-19 Infection UK Cohort	IM	Interleukin-7	25 Years to 80 Years	2	48	2020-05-14
		NCT04379518	Rintatolimod and IFN Alpha-2b for the Treatment of Mild or Moderate COVID-19 Infection in Cancer Patients	IV	Interferon Alfa-2b	18 Years and older	1 & 2	80	2020-05-30
		NCT04357444	Low Dose of IL-2 In Acute Respiratory DistrEss Syndrome Related to COVID-19	SC	ILT101	18 Years and older	2	30	2020-06-01
		NCT04400929	Using GM-CSF as a Host Directed Therapeutic Against COVID-19	IV	Sargramostim	21 Years to 80 Years	2	30	2020-06-01
		NCT04407689	InterLeukin-7 to Improve Clinical Outcomes in Lymphopenic pAtients With COVID-19 Infection FR BL Cohort	IM	Interleukin-7	25 Years to 80 Years	2	48	2020-06-08
		NCT04426201	InterLeukin-7 to Improve Clinical Outcomes in Lymphopenic pAtients With COVID-19 Infection (ILIAD-7-US-O)	IM	CYT107	25 Years to 80 Years	2	48	2020-06-22
		NCT04442178	InterLeukin-7 to Improve Clinical Outcomes in Lymphopenic pAtients With COVID-19 Infection (ILIAD-7-US-I)	IM	CYT107	25 Years to 80 Years	2	48	2020-06-30
		NCT04492475	Adaptive COVID-19 Treatment Trial 3 (ACTT-3)	SC	Interferon beta-1a	18 Years to 99 Years	3	1038	2020-08-04
		NCT04498325	Evaluating the Effect of NT-I7, a Long Acting Interleukin-7, to Increase Lymphocyte Counts and Enhance Immune Clearance of SARS-CoV-2 (COVID-19)	IM	NT-I7	18 Years and older	1	42	2020-09-30
		NCT04810637	A Study to Evaluate the Safety and Efficacy of GX-I7 in Elderly Patients With Asymptomatic or Mild Symptoms of COVID-19	UNS	GX-I7	50 Years and older	2	210	2020-11-01
		NCT04730427	Safety and Preliminary Efficacy Study of GX-I7 in Patients With COVID-19	UNS	GX-I7	19 Years and older	1	40	2021-01-01
		NCT04299724	Safety and Immunity of Covid-19 aAPC Vaccine	SC	APC	6 Months to 80 Years	1	100	2020-02-15
		NCT04324996	A Phase I/II Study of Universal Off-the-shelf NKG2D-ACE2 CAR-NK Cells for Therapy of COVID-19	IV	NK	18 Years and older	1 & 2	90	2020-03-21
		NCT04276896	Immunity and Safety of Covid-19 Synthetic Minigene Vaccine	SC & IV	LV-SMENP-DC vaccine and antigen-specific CTLs	6 Months to 80 Years	1 & 2	100	2020-03-24
		NCT04344548	Phase I / II Clinical Study of Immunotherapy Based on Adoptive Cell Transfer as a Therapeutic Alternative for Patients With COVID-19 in Colombia	IV	NK	18 Years and older	1 & 2	10	2020-04-13
		NCT04365101	Natural Killer Cell (CYNK-001) Infusions in Adults With COVID-19 (CYNK-001-COVID-19)	IV	CYNK-001	18 Years and older	1 & 2	86	2020-05-13
		NCT04386252	Phase Ib-II Trial of Dendritic Cell Vaccine to Prevent COVID-19 in Adults	UNS	AV-COVID-19	18 Years and older	1 & 2	180	2020-07-01

Immune cell	NCT03348670	Follow NCT03305341 - Proof-of-Concept - COVID-19 AP TP Vaccine	IV	CD54+ cells	22 Years to 72 Years	1	20	2020-07-20
	NCT04386252	Phase Ib-II Trial of Dendritic Cell Vaccine to Prevent COVID-19 in Frontline Healthcare Workers and First Responders	SC	AV-COVID-19	18 Years and older	1 & 2	180	2020-09-01
	NCT04578210	Safety Infusion of NatuRal KillEr celLs or MEmory T Cells as Adoptive Therapy in COVID-19 pnEumonia or Lymphopenia	IV	T & NK	up to 80 Years	1 & 2	58	2020-09-04
	NCT04482699	RAPA-501-Allo Off-the-Shelf Therapy of COVID-19	IV	RAPA-501-Allo	18 Years and older	1 & 2	88	2020-09-30
	NCT04401410	Anti-SARS Cov-2 T Cell Infusions for COVID 19	IV	Partially HLA-matched SARS-CoVSTs	18 Years and older	1	58	2020-10-30
	NCT04685603	Dendritic Cell Vaccine to Prevent COVID-19	UNS	AV-COVID-19	18 Years and older	1	27	2020-12-07
	NCT04690387	Dendritic Cell Vaccine, AV-COVID-19, to Prevent COVID-19 Infection	UNS	AV-COVID-19	18 Years and older	1	27	2020-12-07
	NCT04634370	Fase I Clinical Trial on NK Cells for COVID-19	IV	NK	18 Years and older	1	24	2021-01-02
	NCT04765449	Transfer of Infection Fighting Immune Cells Generated in the Laboratory to High Risk Patients With COVID-19 Infection	IV	Cytotoxic T Lymphocytes		1	24	2021-04-30
